# Construction and validation of immune-related LncRNAs classifier to predict prognosis and immunotherapy response in laryngeal squamous cell carcinoma

**DOI:** 10.1186/s12957-022-02608-z

**Published:** 2022-05-24

**Authors:** Xiaofeng Wang, Ya Pan, Yangpeng Ou, Tingting Duan, Yuxia Zou, Xuejun Zhou

**Affiliations:** 1 Department of Otolaryngology-Head and Neck Surgery, the First Affiliated Hospital of Hainan Medical University, Haikou, 570102 Hainan Province People’s Republic of China; 2 Department of Oncology, Huizhou Third People’s Hospital, Guangzhou Medical University, Huizhou, 516000 Guangdong Province People’s Republic of China

**Keywords:** LSCC, IRL classifier, Prognosis, Immune infiltration, Immunotherapy response

## Abstract

**Background:**

Rapid advances in transcriptomic profiles have resulted in recognizing IRLs (immune-related long noncoding RNAs), as modulators of the expression of genes related to immune cells that mediate immune inhibition as well as immune stimulatory, indicating LncRNAs play fundamental roles in immune modulation. Hence, we establish an IRL classifier to precisely predict prognosis and immunotherapeutic efficiency in laryngeal squamous cell carcinoma (LSCC).

**Methods:**

LSCC RNA-seq (RNA sequencing) datasets, somatic mutation data, and corresponding clinicopathologic information were acquired from TCGA (the Cancer Genome Atlas) and Gene Expression Omnibus (GEO) databases. Spearman correlation analysis identified LncRNAs associated with immune-related genes (IRG). Based on Lasso penalized regression and random forest (RF), we constructed an IRL classifier associated with prognosis. GEO database was utilized to validate the IRL classifier. The predictive precision and clinical application of the IRL classifier were assessed and compared to clinicopathologic features. The immune cell infiltration of LSCC was calculated via CIBERSORTx tools and ssGSEA (single-sample gene set enrichment analysis). Then, we systematically correlated the IRL classifier with immunological characteristics from multiple perspectives, such as immune-related cells infiltrating, tumor microenvironment (TME) scoring, microsatellite instability (MSI), tumor mutation burden (TMB), and chemokines. Finally, the TIDE (tumor immune dysfunction and exclusion) algorithm was used to predict response to immunotherapy.

**Results:**

Based on machine learning approach, three prognosis-related IRLs (BARX1-DT, KLHL7-DT, and LINC02154) were selected to build an IRL classifier. The IRL classifier could availably classify patients into the low-risk and high-risk groups based on the different endpoints, including recurrence-free survival (RFS) and overall survival (OS). In terms of predictive ability and clinical utility, the IRL classifier was superior to other clinical characteristics. Encouragingly, similar results were observed in the GEO databases. Immune infiltration analysis displayed immune cells that are significantly richer in low-risk group, CD8 T cells and activated NK cells via CIBERSORTx algorithm as well as activated CD8 T cell via ssGSEA. Additionally, compared with the high-risk group, immune score, CD8 T effector was higher in the low-risk group, yet stromal score, score of p53 signaling pathway and TGFher in the Tx algorithm, was lower in the low-risk group. Corresponding results were confirmed in GEO dataset. Finally, TIDE analysis uncovered that the IRL classifier may be effectually predict the clinical response of immunotherapy in LSCC.

**Conclusion:**

Based on BARX1-DT, KLHL7-DT, and LINC02154, the IRL classifier was established, which can be used to predict the prognosis, immune infiltration status, and immunotherapy response in LSCC patients and might facilitate personalized counseling for immunotherapy.

**Supplementary Information:**

The online version contains supplementary material available at 10.1186/s12957-022-02608-z.

## Introduction

Laryngeal squamous cell carcinoma (LSCC), as an aggressively malignant neoplasm, is by far one of most cancers in respiratory tract as well as head and neck region and accounts for 80–95% of laryngeal cancer [1]. According to the American Cancer Society, about 13,150 cases were diagnosed, with over 3700 deaths annually, and estimated incidence rates were 4.0 per 100,000, with around mortality rates of 1.1 per 100,000 annually [2]. Most patients (~60%) present with advanced-stage disease at diagnosis on account of the lack of obvious symptoms in early stage and apt to lymph node metastasis, which influences the prognosis of patients [3]. Though multidisciplinary and comprehensive therapeutic approaches have been developed, the long-term survival outcome of patients with LSCC is still dismal [4]. Thus, identifying reliable biomarkers and establishing accurate predictive models are urgently necessary to optimize treatment regimens and exploit novel molecular therapies for LSCC patients.

In recent years, immunotherapy, especially the application of ICIs (immune checkpoint inhibitors), has made remarkable progress in antitumor practice and gradually becomes a promising first-line choice in the field of oncology therapy, including LSCC [5]. However, as a heterogeneous disease, LSCC presents conflicting results, with most patients not responding to these inhibitors due to primary or acquired resistance [6, 7]. Unlike conventional therapy, the clinical benefits of immunotherapy to patients are achieved by stimulating the sustained antitumor immune reaction [8], which relies on immunoregulation between cancer cells and TME (tumor microenvironment). Therefore, there is always a need to identify more specific biomarkers, better predictive tools, and screen out which subset of patients with LSCC will respond to these immunotherapies, which may help guide the selection and improvement of effective immunotherapies.

LncRNA, which is ubiquitous in the genome, is a type of noncoding RNA with 200 nucleotides long which cannot encode proteins [9]. Biochemically, LncRNAs exert their function by RNA-protein interactions, RNA-DNA, or RNA-RNA to regulate 70% of human gene expression, which exhibit either enhancement or inhibition [10]. Additionally, it even may account for delivering therapeutic options or prognostic value for neoplasm patients [11]. Recently, increasing evidence has revealed that lncRNAs can regulate the immune response by controlling the homeostasis, TME, anti-inflammatory factors, and functions of immune cells [12, 13]. For example, LncRNA is involved in directing immune cell-specific gene expression, whereby resulting in the alterations of cancer’s immune cell infiltrating. Hence, we ardently anticipate the discovery of several new prognostic IRL (immune-related lncRNA) markers and then build an IRL classifier to precisely predict prognosis and immunotherapeutic efficiency in LSCC.

In the present study, based on machine learning approach, we screen prognosis-related IRLs and then develop an IRL classifier. Subsequently, we estimated the predictive capacity and clinical usefulness of the IRL classifier and compared it against clinicopathologic characteristics. Then, we systematically correlated the IRL classifier with immunological characteristics from multiple perspectives, such as immune-related cells infiltrating, TME scoring, microsatellite instability (MSI), tumor mutation burden (TMB), and chemokines. Finally, TIDE (tumor immune dysfunction and exclusion) algorithm was used to predict response to immunotherapy.

## Materials and methods

### Extraction of public data and data processing

We downloaded RNA-seq data (FPKM value) (111 LSCC tissues and 12 adjacent tissues), somatic mutation data, and corresponding clinicopathologic characteristics of LSCC patients from a public TCGA database (https://gdc.cancer.gov/), which was recorded before December 10, 2021. A total of LC 109 patients were collected with complete follow-up data; the clinical endpoint was recurrence-free survival (RFS) and overall survival (OS). Additionally, three LSCC GEO datasets and matched clinicopathologic information were downloaded, namely GSE65858 (48 LSCC samples), GSE25727 (56 LSCC samples), and GSE27020 (109 LSCC samples) datasets which were used as validation cohorts. The gene expression data were normalized via the Limma package or edgeR package in the R computing environment, which will be further analyzed. Datasets were used according to TCGA and GEO data access strategies. All analyses were carried out in conformity to relevant guidelines and regulations. The IRGs were acquired from gene set M19817 (immune response) and M13664 (immune system process) in MSigDB of Broad Institute (http://www.gsea-msigdb.org/gsea/index.jsp) [14].

### Identification of IRLs and variance analysis

Mining and extraction methods of IRLs were described in previous studies [15]. Based on the expression levels of immune genes and lncRNAs in each specimen, Pearson correlation analysis was conducted using the cor.test function of R (*p*-value < 0.05, correlation coefficient |Cor| > 0.4), and then the cohort of IRLs was identified. To identify the differential expression IRLs (DEIRLs), differential expression analysis was performed in the IRLs cohort via R package Limma. The thresholds were set as FDR (false discovery rate) < 0.05 along with log FC (fold change) > 1.

### Construction and verification of an IRL classifier

First, univariable Cox regression analysis is used to screen prognosis-related IRLs (*p* < 0.05). To select out convincing hub genes, machine learning approach, including modified Lasso penalized regression and RF (random forest), was adopted. A Lasso regression is performed with tenfold cross-validation to identify candidate IRLs and was run for 1000 cycles to select feature variables based on minimum criteria or 1-se criteria. RF (random forest), a tree-based ensemble comprised of tree-structured classifiers, was established to select feature variables via package “randomForest” with minimum error regression trees. The importance of variables was ranked using IncNodePurity. The real hub genes were obtained from the intersection of the result of Lasso and RF, which was used to develop a prediction model, namely IRL classifier.

The IRL score was generated through a linear combination of coefficients from Cox regression and the relative expression of each IRLs. According to this formula, each patient’s IRL score was calculated, and patients were classified into low-risk or high-risk groups on the basis of the median IRL score. Survival differences (log-rank test) were compared by Kaplan-Meier survival analysis between low-risk and high-risk groups based on the different endpoints, including RFS and OS. Time-dependent ROC curves with R package time ROC were adopted to assess predictive performance. Importantly, the GSE65858, GSE27020, and GSE25727 from the GEO database were applied to validate the predictive value of the IRL classifier.

Additionally, according to the expression of individual IRL (BARX1-DT, KLHL7-DT, and LINC02154), patients were classified into low expression or high expression on the basis of the median expression level. Survival differences (log-rank test) were compared by Kaplan-Meier survival analysis between low expression and high expression groups for BARX1-DT, KLHL7-DT, and LINC02154.

### Prognostic significance and clinical application of IRL classifier

Univariate and multivariate Cox regression analyses were applied to investigate whether the predictive capacity of the IRL classifier remains is independent of other clinicopathological features of LSCC patients in TCGA and GEO database. Additionally, ROC analysis using the R package survival ROC was employed to compare the discrimination ability of the IRL classifier against clinicopathological information in TCGA and GEO database. Finally, DCA (decision curve analysis) with the stdca.R package was carried out to estimate the net benefit and clinical usefulness of the IRL classifier and compared to clinicopathological features in TCGA and GEO database [16].

### Evaluation of immune infiltration

ESTIMATE algorithm is a tool to predict the presence of infiltration immune/stromal cells in tumor tissues and tumor purity using gene expression data, which according to single-sample gene set enrichment analysis (ssGSEA) generates ESTIMATE score, immune score, and stromal score.

To evaluate the relative abundance of immune infiltrates, CIBERSORTx (https://cibersort.stanford.edu/) [17], which transformed the normalized gene expression matrix into the composition of infiltrating immune cells, is a kind of deconvolution algorithm with a 1000 permutation count. We filtered out samples with CIBERSORTx output of *p*-value > 0.05 for the accurate forecast of immune cell composition. Then, variance analysis of immune cells between high-risk and low-risk groups was visualized by drawing violin diagrams. In addition, on the basis of the expression of metagenes that are behalf of specific immune cells, the ssGSEA, using R package “GSVA,” was introduced to quantify the relative infiltrating of immune cell subtypes. We focused on the metagene set of 28 immune cell types, which were widely researched and accepted [18].

To determine differential immune cell subtypes between the two groups (*p*-value < 0.05), the Wilcoxon two-tailed test was utilized to analyze the immunoscores. And we adopt vioplotR package to visualize the result. Additionally, we explored the correlation between IRL classifier and critical immune cells by Spearman correlation analyses. A *p <* 0.05 would be considered statistically significant.

### Correlation of IRL classifier with immunological characteristics of the TME

According to the definition of TMB, which is computed using the total covered bases/total number of somatic mutations. Additionally, MSI score was collected from published studies [18]. Correlation analysis was conducted between the IRL classifier and TMB and MSI.

We also investigate the correlation between the IRL classifier and the expression of critical chemokines (CXCL9, CXCL10, and CCR3).

### Cell and cell culture

Human laryngeal carcinoma cells Hep-2 were obtained from BIOBAIYE (Shanghai China). Hep-2 cells were routinely cultured in DMEM (Gibco), which consisted of 10% fetal bovine serum (FBS, Gibco) and 1% penicillin/streptomycin (Solarbio) at 37 °C. Placed within an incubator of 5% CO2, the cells were digested with 0.25% membrane protease (Sigma) every 2–3 days.

### Enzyme-linked immunosorbent assay (ELISA)

To detect the secretion of CXCL9 and CXCL10 in Hep-2 cells, a density of 2 × 10^5^/mL cells was plated. IFN-gamma (PeproTechAsia) was used to stimulate secretion of CXCL9 and CXCL10 in Hep-2 cells. The supernatant was collected after 48 h for analysis using ELISA Kits according to the instructions of the manufacturer (NeoBioscience, China).

### In vitro migration assay

CD8+ T cells were purchased from ATCC. The migration of CD8+ T cells was assessed using 5 μm pore size transwell inserts (Corning Costar). CD8+ T cells were added to the top chamber and culture supernatant from Hep-2 cells after IFN-gamma treatment was added to the bottom chamber. To further confirm the infiltration of CD8+ T cells was influenced by CXCL9 and CXCL10 produced by Hep-2 cells, the culture supernatant from Hep-2 cells was treated with CXCL9 and CXCL10 neutralizing. Then the transwells were incubated at 37 °C, 5% CO2 for 4 h, and cells migrating to the bottom chamber were collected and then counted by a hemocytometer.

### ssGSEA

ssGSEA, generate an enrichment score to signify the levels of absolute enrichment of a metagene set within a given dataset in each sample, was applied to evaluate the enrichment degree of biological processes, including (CD8 T-effector signature, epithelial-mesenchymal transition (EMT) markers including EMT1, EMT2, and EMT3, WNT targets, p53 signaling pathway TGF signaling pathway (EM, and soon [18]) in current immunology research and to compare the differences in enrichment level between high-risk and low-risk subgroups. Additionally, we explored the correlation between IRL classifier and pivotal molecular pathways by Spearman correlation analyses. A *p <* 0.05 would be considered statistically significant.

### TIDE

The TIDE method (http://tide.dfci.harvard.edu), which was considered a reliable algorithm to predict the immunotherapeutic response of patients (CTLA-4 and PD inhibitor) in recent research [19], was used to evaluate the predictive efficiency of IRL classifier for the ICIs response in LSCC. On the basis of the TIDE value, a TIDE score less than 0 was recognized as positive sensitivity to immunotherapy (a patient as a responder), while a TIDE score more than 0 was considered as negative sensitivity to immunotherapy (a patient as a nonresponder). We compare the rate of response between high-risk and low-risk groups.

### Statistical analysis

SPSS statistics 22.0 and R software (R version 3.6.1) were used to perform the statistical analysis. A *p <* 0.05 (two-sided) was considered statistically significant unless otherwise agreed. Entire R codes were provided in Supplementary material 1.

## Results

### Identification of IRLs and variance analysis

Altogether, 331 immune-related genes were acquired in MSigDB of Broad Institute, and 12830 lncRNAs were obtained from LSCC cohort. Immune-related gene and the lncRNA coexpression network were assembled to visualize these IRLs. Ultimately, 5192 IRLs were identified in our research with *p*-value < 0.05 and correlation coefficient |Cor| > 0.4 (Supplementary material 2). The difference analysis screened 486 DEIRLs between normal and tumor samples, which was subjected to univariable Cox regression analysis.

### Construction and verification of an IRL classifier

**Fig. 1:**
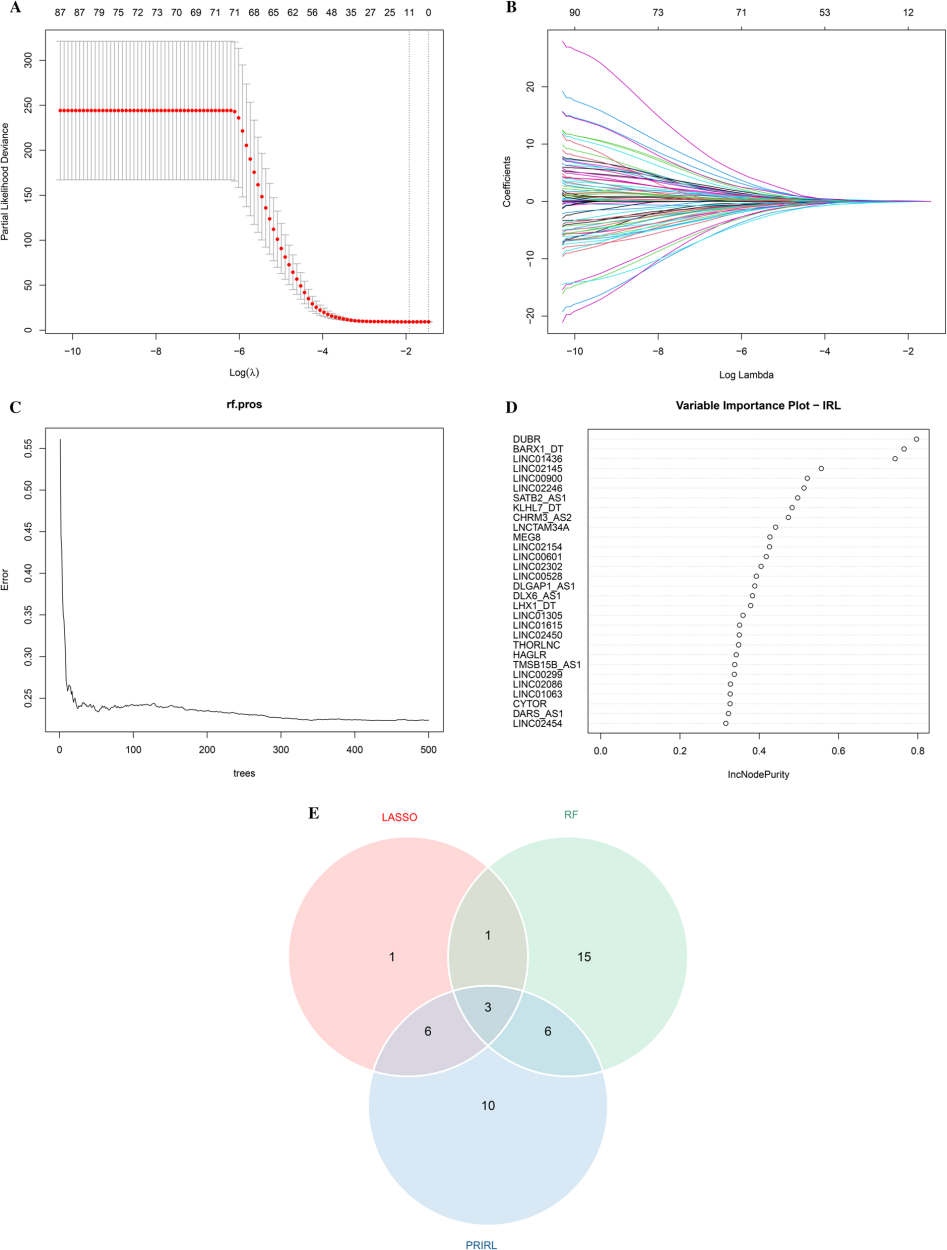
IRLs selected by Lasso regression analysis and random forest (RF). **A** The two dotted vertical lines are drawn at the optimal values by minimum criteria (right) and 1-se criteria (left). **B** Lasso coefficient profiles of the 11 IRLs. A vertical line is drawn at the optimal value by 1-se criteria and results in 11 nonzero coefficients. **C** Distribution diagram of regression tree and error. **D** The top 15 most important variables ranked by IncNodePurity. **E** Venn diagram presents the intersection of three results to identify hub genes

By univariable Cox regression analysis, we appraised 101 prognostic-related IRLs (Supplementary material 3). Modified Lasso penalized regression was established to shrink and select out hub IRLs, as shown in Fig. 1A and B. Likewise, RF was also built with minimum error regression trees for hub IRLs (Fig. 1C and D). According to the result of Lasso, RF, and top 25 prognostic-related IRLs, we take the intersection of three results to acquire 3 hub genes (BARX1-DT, KLHL7-DT, and LINC02154) (Fig. 1E). Subsequently, the 3 hub genes were used to develop a prediction model, namely IRL classifier. The IRL classifier risk score was calculated as follows: IRL score = (0.1882 expression level of BARX1-DT) + (0.3130 expression level of KLHL7-DT) + (0.2296 expression level of LINC02154). We computed each sample risk score and divided patients with LSCC into a high-risk cohort and a low-risk cohort in TCGA and GEO datasets.

**Fig. 2:**
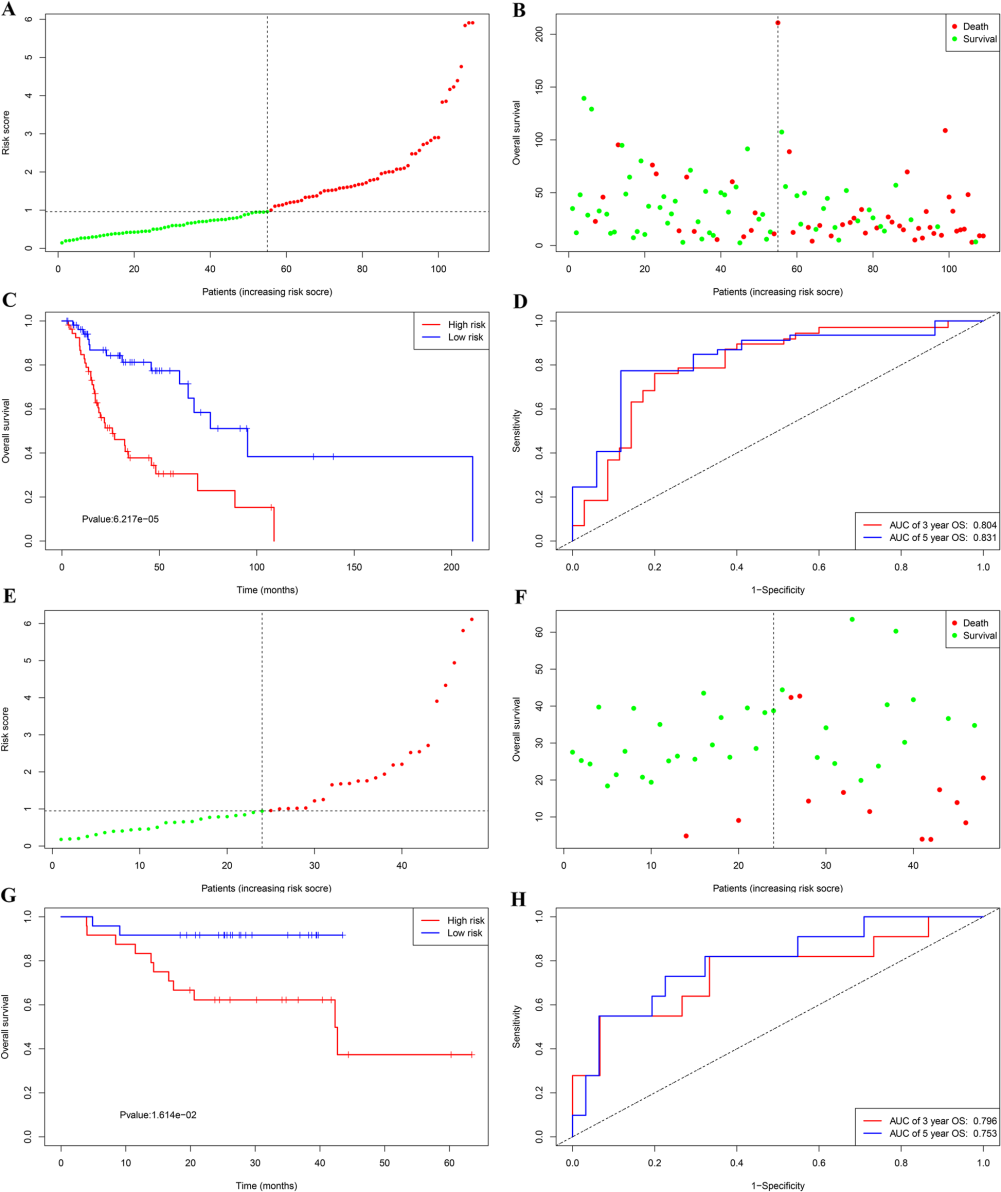
Development of IRL classifier for prediction of OS in LSCC patients in TCGA and GSE65858 database. **A** and **B** Distribution of IRL score in TCGA database. **C** Time-independent ROC curves with AUC values to evaluate predictive efficacy of IRL score in TCGA database. **D** Kaplan-Meier estimates of patients’ survival status and time using the median risk score cutoff which divided patients into low-risk and high-risk groups in TCGA database. **E** and **F** Distribution of IRL score in GSE65858 database. **G** Time-independent ROC curves with AUC values to evaluate predictive efficacy of IRL score in GSE65858 database. **H** Kaplan-Meier estimates of patients’ survival status and time using the median risk score cutoff which divided patients into low-risk and high-risk groups in GSE65858 database

According to the median value of the IRL score (Fig. 2A), intuitively, there is a higher death rate in high-risk cohorts than in low-risk cohorts (Fig. 2B). The Kaplan-Meier curves displayed that the OS (*p*-value < 0.001) of the low-risk groups was significantly higher than that of the high-risk groups, demonstrating the effectiveness of the IRL classifier (Fig. 2C). Time-dependent ROC curves displayed that IRL classifier had a superior prediction ability, with an AUC of 0.831 (5 years) and AUC of 0.804 (3 years) for OS (Fig. 2D). Additionally, external GEO cohorts (GSE65858 database) were utilized to verify the predictive performance of the IRL classifier. As was displayed in Fig. 2E–H, patients with low-risk score were more prone to survival and had higher OS time than patients with high-risk score, which is consistent with the results of the TCGA dataset. Furthermore, the AUC of IRL classifier (AUC of 5-year OS: 0.753 and AUC 3-year OS: 0.796 in GSE65858 database) confirmed that the predictive accuracy of the prognostic model was satisfactory.

**Fig. 3:**
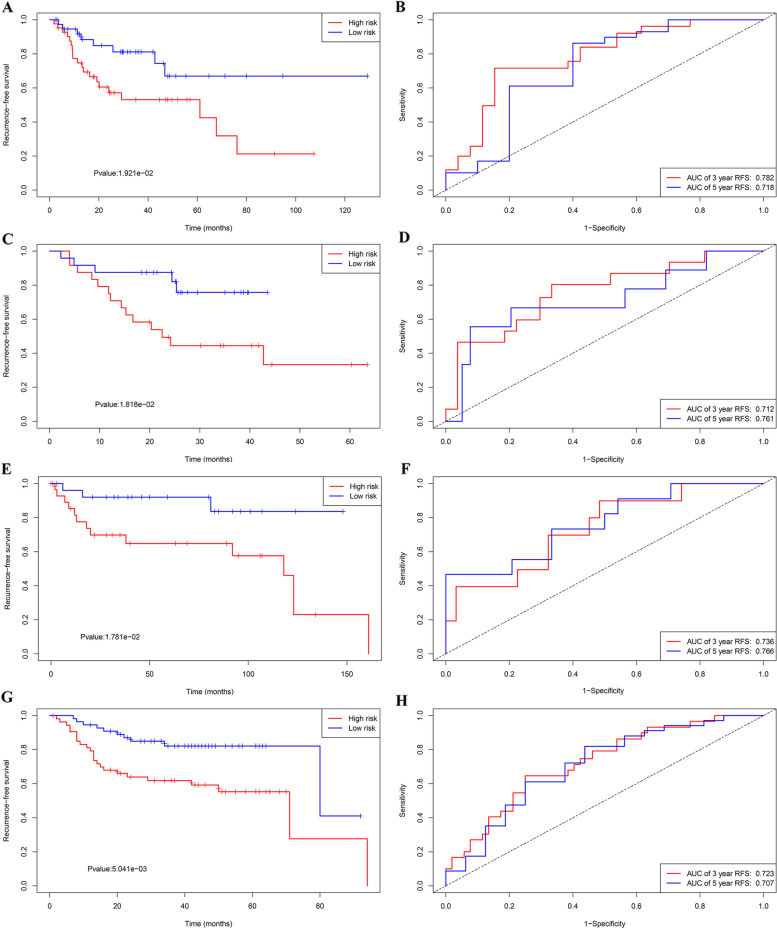
Development of IRL classifier for prediction of RFS/PFS in LSCC patients in TCGA and GEO database. **A** Kaplan-Meier curves of RFS analysis for IRL classifier in TCGA database. **B** Time-independent ROC curves with AUC values to evaluate predictive efficacy of IRL score in TCGA database. **C** Kaplan-Meier curves of PFS analysis for IRL classifier in GSE65858 database. **D** Time-independent ROC curves with AUC values to evaluate predictive efficacy of IRL score in GSE65858 database. **E** Kaplan-Meier curves of RFS analysis for IRL classifier in GSE25727 database. **F** Time-independent ROC curves with AUC values to evaluate predictive efficacy of IRL score in GSE25727 database. **G** Kaplan-Meier curves of RFS analysis for IRL classifier in GSE27020 database. **H** Time-independent ROC curves with AUC values to evaluate predictive efficacy of IRL score in GSE27020 database

We also assessed the ability of IRL classifier to predict the relapse/progression in patients with LSCC. In the TCGA cohort, the Kaplan-Meier survival curves indicated that high-risk cohorts had significantly worse RFS compared with patients with high-risk cohorts (Fig. 3A). Time-dependent ROC curves displayed that IRL classifier had an excellent prediction ability, with an AUC of 0.782 (5 years) and AUC of 0.718 (3-year) for RFS (Fig. 3B). Consistent results were observed in GEO cohorts (GSE65858, GSE27020, and GSE25727 databases). As was shown in Fig. 3C–H, patients with low-risk score were more prone to relapse/progression with higher RFS/progression-free survival (PFS) time than patients with high-risk score. Furthermore, the AUC of IRL classifier (AUC of 5-year PFS: 0.761 and AUC 3-year PFS: 0.712 in GSE65858 database, AUC of 5-year RFS: 0.736 and AUC 3-year RFS: 0.766 in GSE25727 dataset, AUC of 5-year RFS: 0.707 and AUC 3-year RFS: 0.723 in GSE27020 dataset) verified that the predictive accuracy of the IRL classifier was satisfactory.

The K-M curves and the log-rank test showed that patients in the high-BARX1-DT group had worse OS than the patients in the low group (Fig. S1A, *p*-value < 0.001), patients in the high-KLHL7-DT group also had worse OS compared with the patients in the low group (Fig. S1B, *p*-value = 0.048), and high expression of LINC02154 had significantly worse OS compared to patients with low expression of LINC02154 (Fig. S1C, *p*-value = 0.010) in the TCGA cohort. Similar results were observed in GEO cohorts, except for LINC02154 (Fig. S1D–F).

### Prognostic significance and clinical application of IRL classifier

To adjudicate whether the IRL classifier is independent of the clinical features, univariate and multivariate Cox regression analysis uncovered that IRL score (*p*-value < 0.05) was an independent predictor of poor prognosis, in spite of other clinicopathologic characteristics in TCGA (Table 1) and GEO datasets (Table 2).

**Table 1:** Univariable and multivariable Cox regression analysis for prediction of survival in TCGA database

Factors	Subgroup	Univariable analysis		Multivariable analysis	
		HR (95% *CI*)	***P***	HR (95% *CI*)	***P***
**Age**		1.00 (0.97–1.04)	0.984	NA	NA
**Sex**	Female	1			
	Male	0.28 (0.14–0.56)	0.000*	0.31 (0.11–0.88)	0.028*
**Smoking history**	No	1			
	Yes	0.65 (0.35–1.18)	0.156	NA	NA
**Alcohol history**	No	1			
	Yes	0.77 (0.43–1.39)	0.388	NA	NA
**Number of lymph nodes**		1.00 (0.98–1.01)	0.558	NA	NA
**Number of positive LNs**		1.00 (0.95–1.04)	0.892	NA	NA
**Lymph node ratio**		1.41 (0.28–7.13)	0.675	NA	NA
**Margin status**	Negative	1		1	
	Positive	4.68 (2.08–10.51)	0.000*	2.89 (0.97–8.62)	0.058
**Lymphovascular**	No	1		1	
**Invasion**	Yes	4.10 (2.23–7.55)	0.000*	1.62 (0.70–3.62)	0.262
**Tumor grade**	G1-G2	1			
	G3-G4	0.51 (0.25–1.04)	0.064	NA	NA
**Clinical T**	T1-T2	1			
	T3-T4	0.72 (0.35–1.50)	0.376	NA	NA
**Clinical N**	N0	1			
	N1-N3	1.44 (0.80–2.57)	0.222	NA	NA
**Clinical stage**	I-II	1			
	III-IV	0.86 (0.36–2.03)	0.729	NA	NA
**Mutation count**		0.99 (0.98–1.01)	0.542	NA	NA
**Fraction genome altered**		1.44 (0.29–7.18)	0.654	NA	NA
**IRL score**		1.78 (1.46–2.17)	0.000*	1.57 (1.13–2.20)	0.007*

These variables were eliminated in the multivariate cox regression model, so the HR and *p*-values were not available

*HR* hazard ratio, *CI* confidence intervals, *NA* not available

*p < 0.05

**Table 2:** Univariable and multivariable Cox regression analysis for prediction of survival in GEO database

Dataset	Factors	Subgroup	Univariable analysis		Multivariable analysis	
			HR (95% *CI*)	***P***	HR (95% *CI*)	***P***
**GSE65858**	**Age**		0.92 (0.86–0.99)	0.024*	0.98 (0.92–1.05)	0.536
	**Sex**	Female	1			
		Male	0.28 (0.14–0.56)	0.000*	0.28 (0.03–2.82)	0.280
	**Smoking history**	No	1			
		Yes	0.29 (0.02–3.92)	0.348	NA	NA
	**Alcohol history**	No	1			
		Yes	0.74 (0.12–4.55)	0.743	NA	NA
	**Pack years**		1.02 (0.97–1.06)	0.457	NA	NA
	**HPV status**	Negative	1			
		Positive	0.12 (0.08–35.24)	0.933	NA	NA
	**Clinical T**	T1-T2	1			
		T3-T4	1.12 (0.36–3.52)	0.952	NA	NA
	**Clinical N**	N0	1			
		N1-N3	1.31 (0.80–2.12)	0.514	NA	NA
	**Clinical stage**	I-II	1			
		III-IV	1.06 (0.72–2.54)	0.952	NA	NA
	**Treatment modalities**	Mono-treatment	1			
		Multi-treatment	1.44 (0.29–7.18)	0.654	NA	NA
	**IRL score**		1.32 (1.06–1.74)	0.003*	1.6 8 (1.44–2.42)	0.004*
**GSE27020**	**Age**		1.02 (0.99–1.06)	0.193	NA	NA
	**Sex**	Female	1			
		Male	21.94 (0.34–32.16)	0.377	NA	NA
	**Smoking history**	No	1			
		Yes	20.53 (0.06–28.44)	0.679	NA	NA
	**Alcohol history**	No	1			
		Yes	1.17 (0.58–2.33)	0.664	NA	NA
	**Tumor grade**	G1-G2	1			
		G3-G4	0.90 (0.64–1.26)	0.998	NA	NA
	**Clinical stage**	I-II	1			
		III-IV	1.00 (0.61–1.65)	0.525	NA	NA
	**Radiation therapy**	Yes			NA	NA
		No	2.22 (0.98–4.99)	0.055	NA	NA
	**IRL score**		1.92 (1.42–2.61)	0.000*	1.92 (1.42–2.61)	0.000*

These variables were eliminated in the multivariate cox regression model, so the HR and *p*-values were not available

*HR* hazard ratio, *CI* confidence intervals, *NA* not available

*p < 0.05

**Fig. 4:**
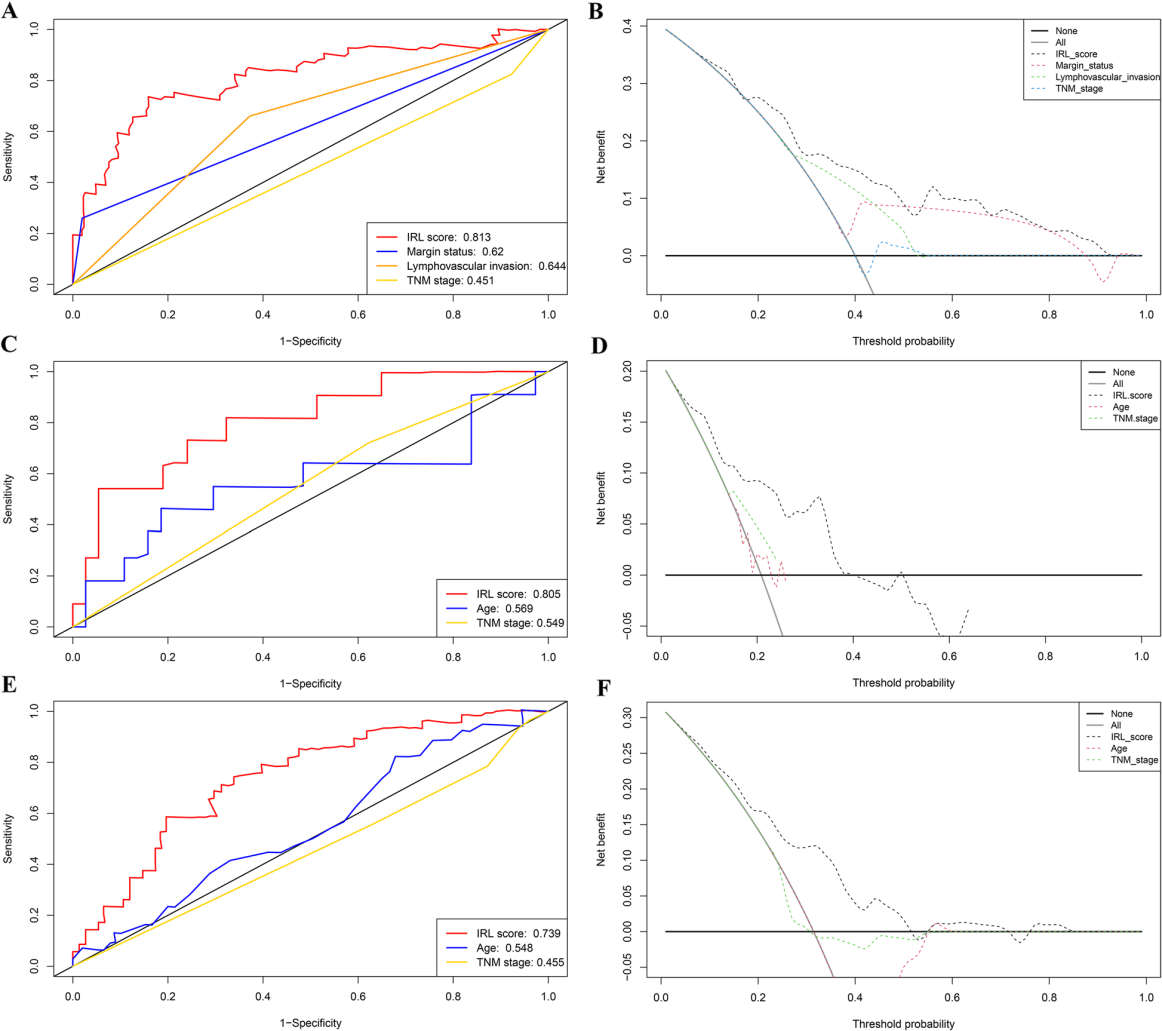
The predictive precision and clinical application of the IRL classifier were assessed and compared to clinicopathologic features. **A** ROC curves in TCGA database. **B** DCA in TCGA database. **C** ROC curves in GSE65858 database. **D** DCA in GSE65858 database. **E** ROC curves in GSE27020 database. **F** DCA in GSE27020 database

In addition, to evaluate the predictive capacity of IRL classifier, IRL classifier (AUC: 0.813) performed better in predicting prognosis than margin status (AUC: 0.62), lymphovascular invasion (AUC: 0.644), and TNM stage (AUC: 0.451) in TCGA datasets (Fig. 4A); IRL classifier (AUC: 0.805) performed better in predicting prognosis than age (AUC: 0.569), sex (AUC: 0.421), and TNM stage (AUC: 0.549) in GSE65858 datasets (Fig. 4C). IRL classifier (AUC: 0.739) performed better in predicting prognosis than age (AUC: 0.548) and TNM stage (AUC: 0.455) in GSE27020 datasets (Fig. 4E). Notably, DCA chart shows that the IRL classifier outperforms age, margin status, lymphovascular invasion, and TNM stage according to the net benefit of risk stratification using the model (y-axis) and the continuity of potential death threshold (x-axis) in TCGA and GEO cohorts (Fig. 4B, D, and F).

### Estimation of immune infiltration

Based on the ESTIMATE algorithm, compared with the low-risk group, the immune score (*p*-value < 0.05) of the high-risk group was lower, yet the stromal score (*p*-value < 0.05) of the high-risk group was higher (Fig. 6A and B), while ESTIMATE score did not exert statistical difference (Fig. 6C). Similar results were observed in GEO cohorts (Fig. 6D–F).

**Fig. 5:**
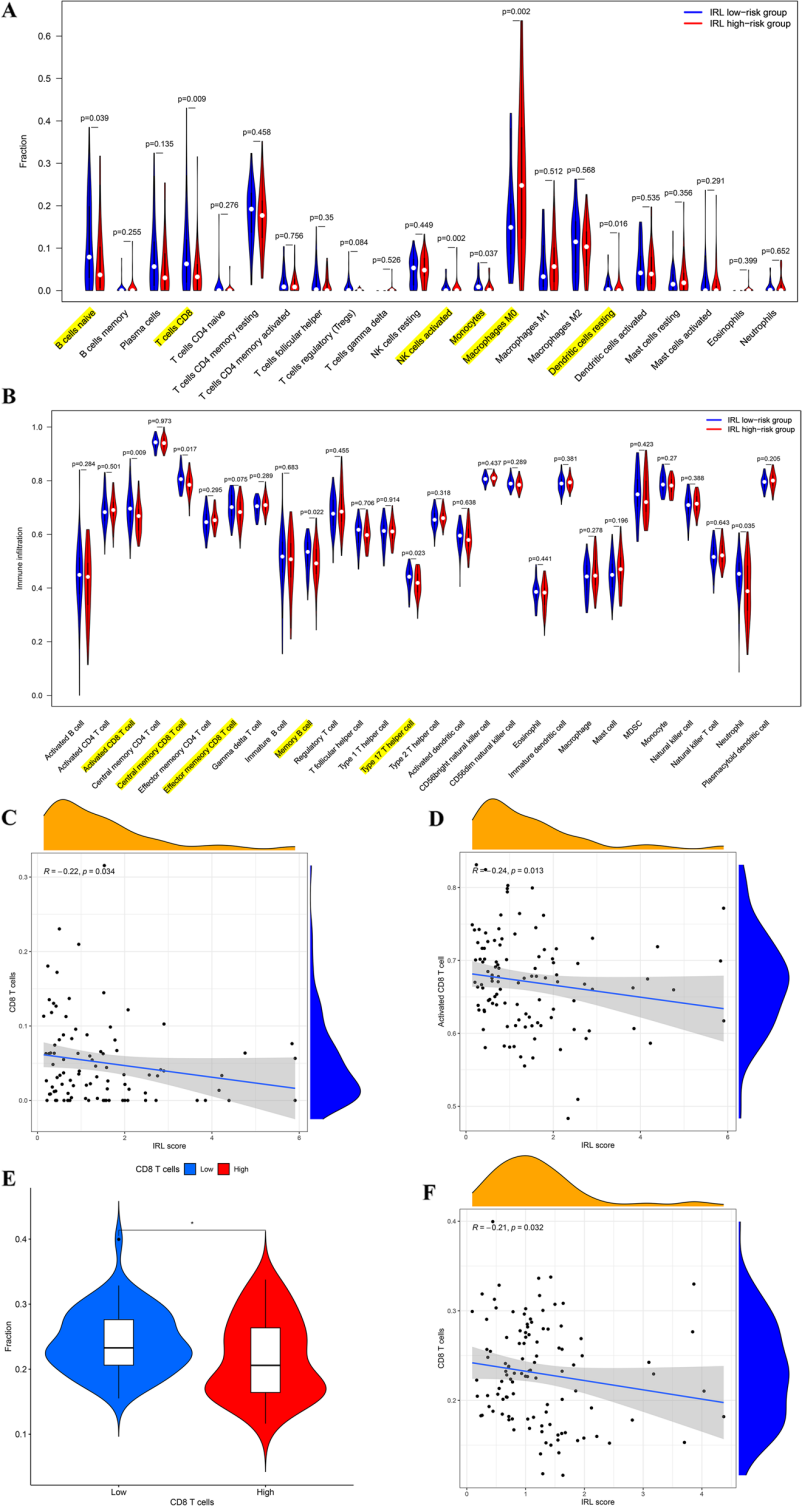
Analyzing of infiltrating immune cells between high-risk group and low-risk group. **A** CIBERSORTx tool in TCGA database. **B** ssGSEA in TCGA database. **C** Correlation between IRL classifier and CD8 T cells in TCGA database. **D** Correlation between IRL classifier and activated CD8 T cells in TCGA database. **E** Comparison of CD8 T cells between IRL different subgroups in GEO database. **F** Correlation between IRL classifier and CD8 T cells in GEO database

We investigate the difference in infiltrating immune cells between the two groups; the CIBERSORTx results demonstrate that compared with high-risk group, CD8 T cells (*p* = 0.009) and activated NK cells (*p* = 0.002) and so on were more abundant in low-risk group (Fig. 5A). In addition, the ssGSEA results uncover that compared with high-risk group, activated CD8 T cells (*p* = 0.009) and Th17 cells (*p* = 0.023) were significantly higher in low-risk groups (Fig. 5B). This finding reveals that CD8 T cells were significantly richer in low-risk group.

IRL score was significantly negatively correlated with CD8 T cells (*R* = −0.22, *p* = 0.034) (Fig. 5C) and activated CD8 T cells (*R* = −0.24, *p* = 0.013) (Fig. 5D). Importantly, compared to high-risk group, CD8 T cells (*p*-value < 0.05) were more abundant in low-risk group (Fig. 5E), and IRL score was significantly negatively associated with CD8 T cells (*R* = −0.21, *p* = 0.032) (Fig. 5F) in GEO dataset, which line with the results of the TCGA dataset.

### Correlation IRL classifier with immunological characteristics of the TME

**Fig. 6:**
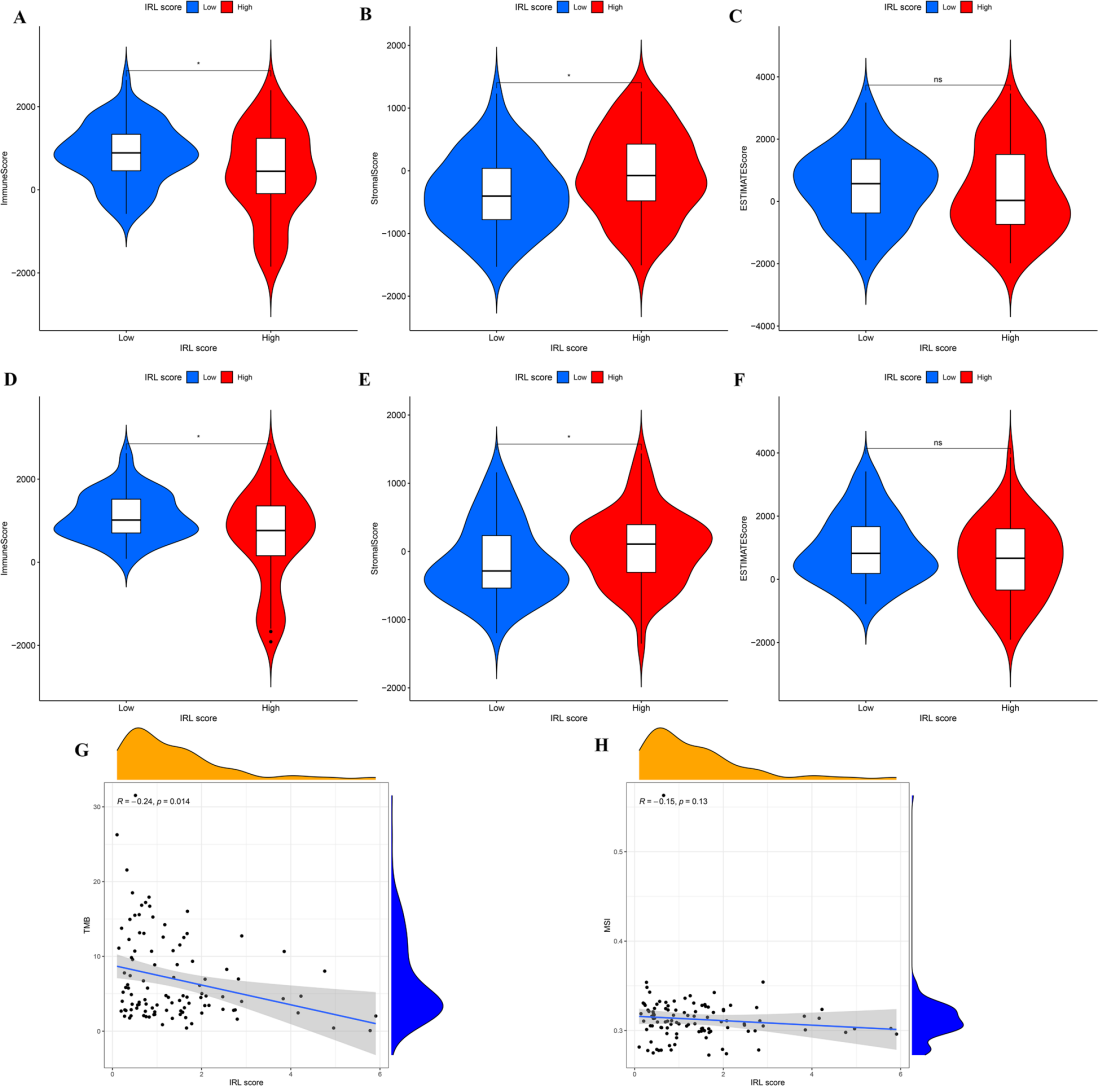
The comparison of the microenvironment score between IRL different subgroups in TCGA and GEO database. **A** Immune score in TCGA database. **B** Stromal score in TCGA database. **C** ESTIMATE score in TCGA database. **D** Immune score in GEO database. **E** Stromal score in GEO database. **F** ESTIMATE score in GEO database. **G** Correlation between IRL score and TMB. **H** Correlation between IRL score and MSI

**Fig. 7:**
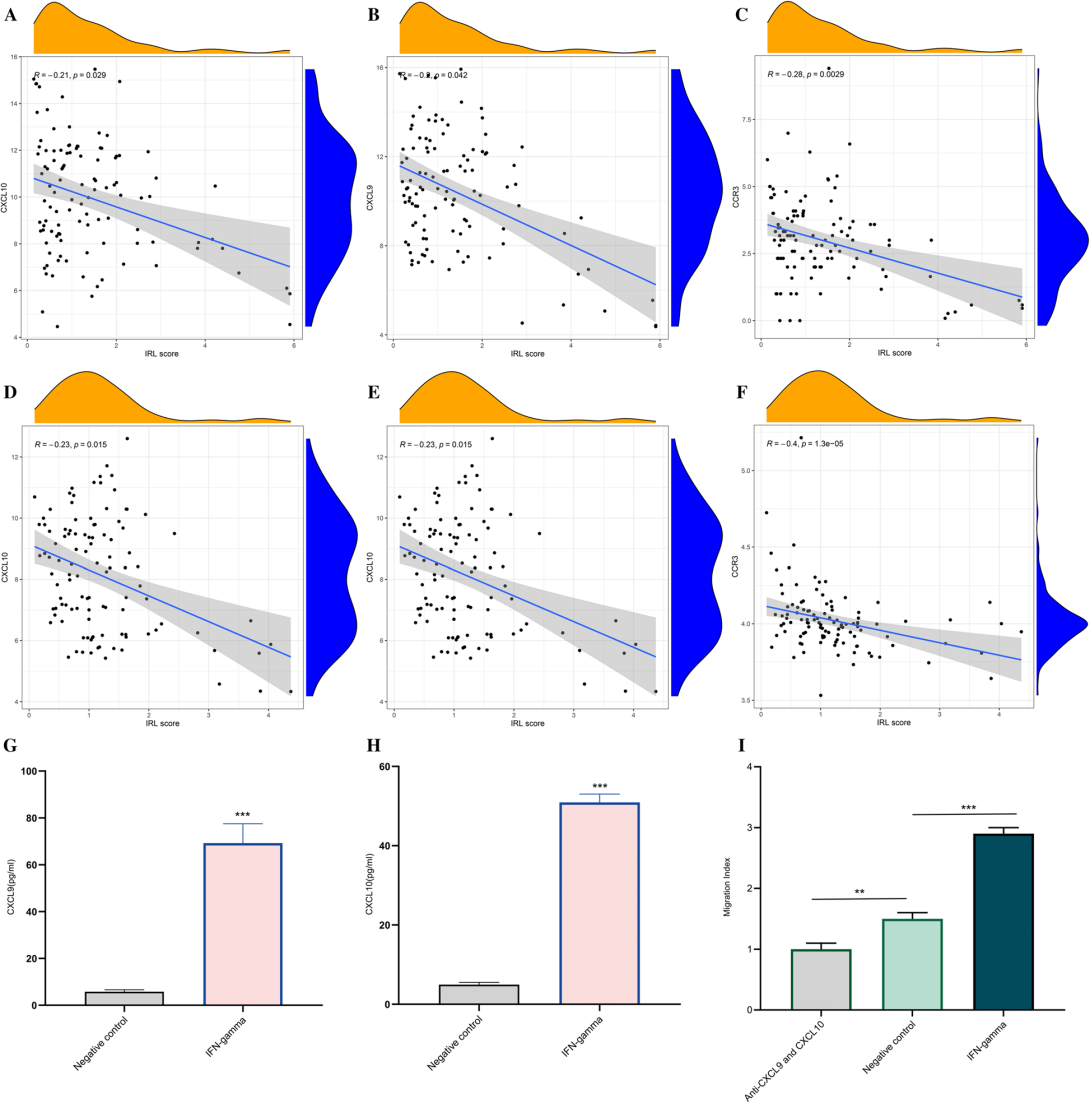
Correlation between IRL score and three critical chemokines in LSCC. **A** CXCL9 in TCGA database. **B** CXCL10 in TCGA database. **C** CCR3 in TCGA database. **D** CXCL9 in GEO database. **E** CXCL10 in GEO database. **F** CCR3 in GEO database. **G** The expression of CXCL9 in the supernatant of Hep-2 after IFN-gramma cells and negative control Hep-2 cells detected by ELISA assay. **H** The expression of CXCL9 in the supernatant of Hep-2 after IFN-gramma cells and negative control Hep-2 cells detected by ELISA assay. **I** Migration of CD8+ T cells towards supernatants of Hep-2 after IFN-gramma cells, negative control Hep-2 cells, and anti-CXCL9 and CXCL10 were detected utilizing the transwell assay. *p < 0.05, **p ≤ 0.01, and ***p ≤ 0.001

We explore the relationship between tumor immunogenicity and IRL score. As a result, the IRL score was significantly negatively correlated with TMB (*R* = −0.24, *p* = 0.014) (Fig. 6G), while the IRL score was not significantly correlated with MSI (Fig. 6H). We investigate the potential cause of CD8 T cells enriched in IRL low-risk group; as a result, IRL score was significantly negatively associated with CD8 T cells the expression of critical chemokines (CXCL9, CXCL10, and CCR3) in TCGA and GEO datasets (Fig. 7A–F).

### ELISA

We detected the level of CXCL9 and CXCL10 in Hep-2 cells after IFN-gamma treatment and negative control Hep-2 cells derived supernatants. ELISA results showed that the secretion of CXCL9 (Fig. 7G) and CXCL10 (Fig. 7H) in IFN-gamma group was significantly upregulated than negative control group.

### In vitro migration assay

Further analysis evaluating the CXCL9 and CXCL10 as well as CD8+ T cell recruitment in LSCC was performed using transwell migration assays in vitro.

The supernatant of Hep-2 cells after IFN-gamma treatment cells induced more CD8+ T-cell migration in comparison with that of negative control, while the CCL3 and CCL20 neutralizing antibodies significantly inhibited the CD8+ T cells chemotaxis ability (Fig. 7I).

### ssGSEA

**Fig. 8:**
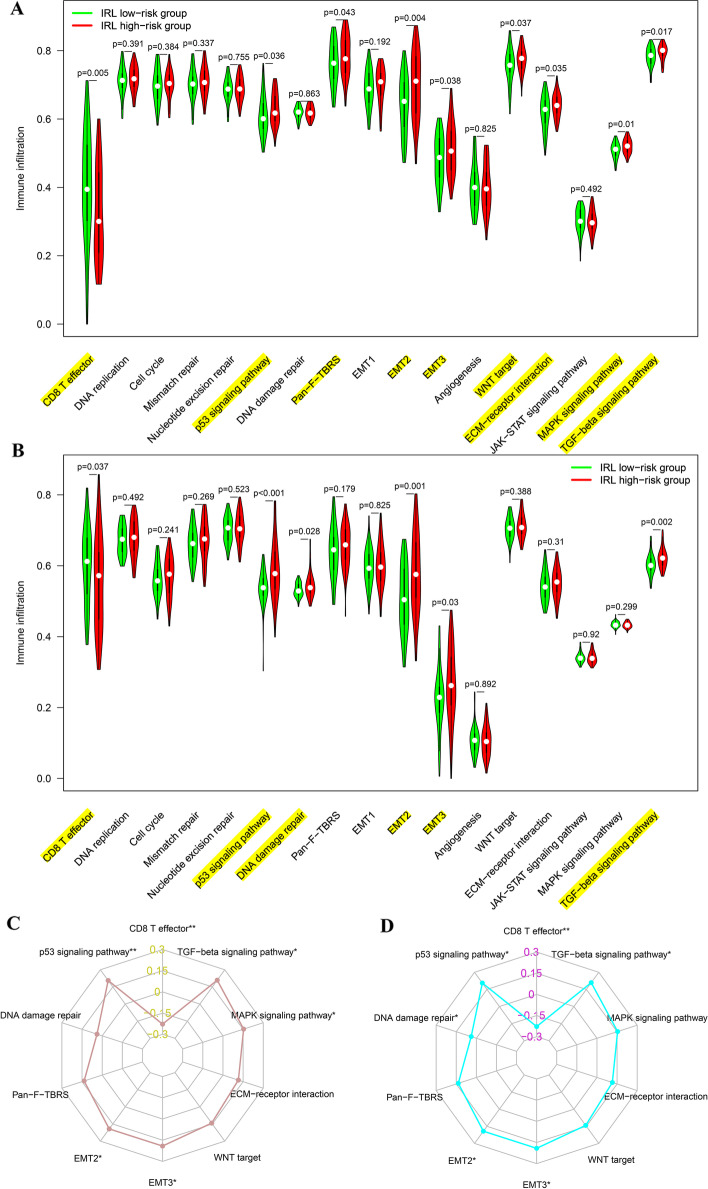
Comparison of biological pathway between IRL different subgroups in TCGA and GEO database. **A** TCGA database. **B** GEO database. **C** The radar plot in TCGA database. **D** The radar plot in GEO database

To investigate potential biological pathways between high-risk and low-risk groups, we carried out ssGSEA to explore the predefine pathway in LSCC. In TCGA cohorts, the ssGSEA results showed that CD8 T effector was significantly involved in the low-risk group, while p53 signaling pathway, EMT2, EMT3, and TGF-beta signaling pathway were significantly enriched in the high-risk group (Fig. 8A). Similar results were found in GEO cohorts (Fig. 8B). As shown in the radar plot (Fig. 8C and D), IRL score was significantly negatively correlated with CD8 T effector while was significantly positively associated with p53 signaling pathway, EMT2, EMT3, and TGF-beta signaling pathway in TCGA and GEO datasets.

### Immunotherapeutic prediction of IRL classifier

**Fig. 9:**
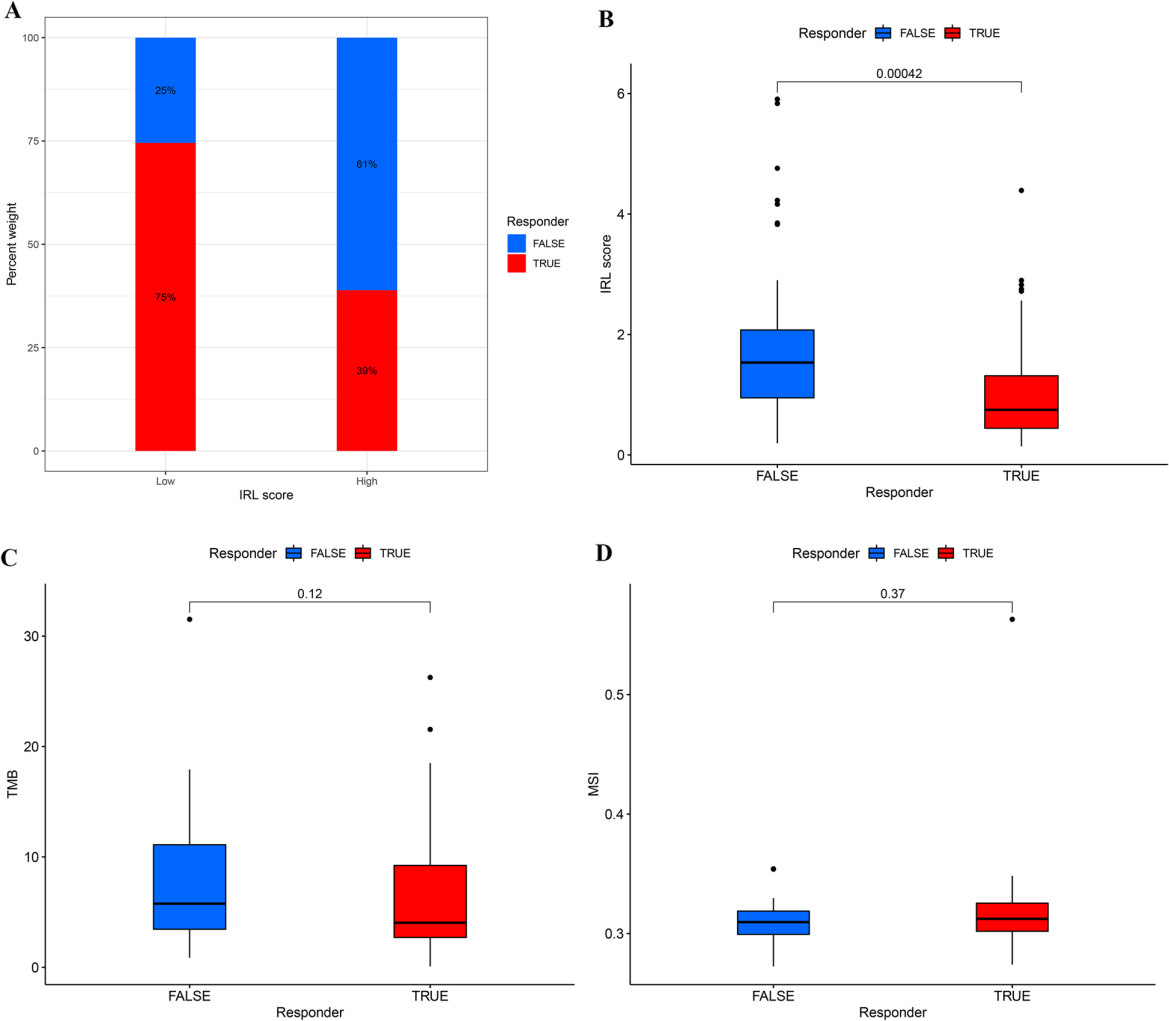
**A** The distribution of immunotherapeutic response in two groups stratified by IRL classifier in LSCC cohort based on the TIDE algorithm. Two-sided chi-square test was used to analyze contingency tables for ICIs responder. **B** Comparison of IRL score between responder group and non-responder group. **C** Comparison of TMB between responder group and non-responder group. **D** Comparison of MSI between responder group and non-responder group

We applied TIDE to evaluate the potential clinical efficacy of immunotherapy in different IRL groups. The result uncovered that the number of immunotherapeutic responders was significantly higher in low-risk groups (41/55) compared to high-risk groups (13/54) (two-sided chi-square test, *p*-value < 0.001) (Fig. 9A). In addition, compared to responders, nonresponders had a higher IRL score (*p*-value < 0.001) (Fig. 9B), while TMB (*p* = 0.12), and MSI (*p* = 0.37) did not exert statistical difference between responders and nonresponders (Fig. 9C and D).

## Discussion

LSCC TCGA datasets were utilized as discovery cohorts, and three LSCC GEO datasets were used as validation cohorts. Based on machine learning approach, three prognosis-related IRLs (BARX1-DT, KLHL7-DT, and LINC02154) were selected to build an IRL classifier. The IRL classifier could availably classify patients into the low-risk and high-risk groups based on the different endpoints, including recurrence-free survival (RFS) and overall survival (OS). In terms of predictive ability and clinical utility, the IRL classifier was superior to other clinical characteristics. Encouragingly, similar results were observed in the GEO databases. Immune infiltration analysis displayed immune cells that are significantly richer in low-risk group, CD8 T cells and activated NK cells via CIBERSORTx algorithm as well as activated CD8 T cell via ssGSEA. Additionally, compared with the high-risk group, immune score, CD8 T effector was higher in the low-risk group, yet stromal score, score of p53 signaling pathway and TGF−beta signaling pathway, was lower in the low-risk group. Corresponding results were confirmed in GEO dataset. IRL score is significantly negatively correlated with TMB, but not with MSI. Finally, TIDE analysis uncovered that the IRL classifier maybe effectually predict the clinical response of immunotherapy in LSCC.

An increasing body of evidence indicates that IRLs, as modulators of the expression of genes related to immune cell that mediates immune inhibition as well as immune stimulatory, are involved in the TME, the differentiation of immune cell, and cancer immunity cycle [20–23]. NcRNA-RB1 (a lncRNA expressed in the RB1 promoter) suppresses the expression of calreticulin, which is a calcium-binding chaperone and affects the presentation of antigen to cytotoxic T cells, and prevents tumor cells release “killing me” signal [20]. Pei et al. (2018) indicated that the interaction of small nucleolar RNA host gene 1 (LncRNA-SNHG1) with miR-448 negatively regulates the protein level of IDO to inhibit Treg cells differentiation in circulating peripheral blood and impede immune escape [21]. In vivo experiments exhibited that the suppression of NEAT1 (a nuclear paraspeckle localized LncRNA) by the miR-155/Tim-3 pathway reduces CD8+ T-cell apoptosis and enhances active cytolytic function, thereby achieving immune activity [22, 23]. In addition, Tim-3 upregulated in chronic infection as well as in exhausted T cells in tumors. In turn, the increased Tim-3 expression results in CD8+ T-cell death. These studies indicated that IRLs participate in the modulators of immune response and the cancer immunity cycle. Currently, immunotherapy targeting ICIs have been applied in the clinical trial of advanced LSCC, yet most patients did not respond to these inhibitors. Thus, it is necessary to investigate novel prospective prognostic IRLs markers, which may be useful for guiding the selection and improvement of immunotherapy.

To our knowledge, this is the first study conducted to identify new IRLs, establish an IRL classifier to precisely predict prognosis, and then comprehensively investigate the IRL classifier correlated with immunological characteristics from multiple perspectives, such as immune-related cells infiltrating, tumor microenvironment (TME) scoring, microsatellite instability (MSI), tumor mutation burden(TMB), and chemokines, thus analyzing immunotherapy response in LSCC.

Based on RF and Lasso, which were applied to identify reliable feature variables, 3 IRLs (BARX1-DT, KLHL7-DT, and LINC02154) were identified as hub genes, which were combined to construct an IRL classifier. It can effectually divide patients into low-risk group with longer survival and high-risk group with shorter survival based on the different endpoints, including RFS and OS. Additionally, external GEO database (GSE65858, GSE27020, and GSE25727 datasets) was utilized to verify the predictive performance of the IRL classifier. As a result, patients with low-risk score were more prone to survival and had higher OS/RFS time than patients with high-risk score, which is consistent with the results of the TCGA dataset. Furthermore, the higher AUC of IRL classifier in multiple transcriptome sets confirmed that the predictive accuracy of the prognostic model was satisfactory.

Univariate and multivariate Cox analysis verified that IRL classifier was an independent predictor of poor prognosis in multi-transcriptome datasets, regardless of other clinical features, which indicated that IRL classifier was a robust risk model. Additionally, the performance of the IRL classifier in predicting mortality outcomes is superior to clinical features. Importantly, DCA results demonstrated that survival-associated treatment decisions for LSCC patients based on the IRL classifier had a net benefit compared to treatment decisions based on other clinical features or treatment for all patients or none. To sum up, the current IRL classifier will be helpful for clinicians to tailor survival-related treatment decisions.

The proportion and number of immune cells infiltration in the TME are considered important elements influencing cancer progression and immunotherapy response. According to the ESTIMATE algorithm, compared to the high-risk group, the immune score of the low-risk group was higher, which indicated that low-risk group is in a state of immune activation. Additionally, the CIBERSORTx tool and ssGSEA algorithm were used for the first time to analyze the immune cell infiltration landscape in LSCC. The CIBERSORTx results demonstrate that compared with high-risk group, CD8 T cells and activated NK cells were more abundant in low-risk group. Analogously, the ssGSEA results uncover that compared to high-risk group, activated CD8 T cells and Th17 cells were significantly richer in low-risk group. This finding indicates that immune-activated cells, such as CD8 T cells [24], were significantly richer in low-risk group, which can effectively recognize antigens to kill tumor cells and enhance the effect of ICI immunotherapy. Since chemokines and chemokine receptors induce the recruitment of multiple immune cells into the TME, including the movement of CD8 positive T cells to enhance immune infiltration and antitumor immunity [25], we found that IRL classifier was markedly negatively related to CCR3, CXCL10, and CXCL9 expression in LSCC tissues. Importantly, in vitro migration assay verified that the secretion of CXCL9 and CXCL10 can promote CD8+ T-cell recruitment in LSCC. Thus, we speculate that 3 IRLs (BARX1-DT, KLHL7-DT, and LINC02154) may boost the development of an immunosuppressive TME by thoroughly downregulating the expression of key immunomodulators such as CCR3, CXCL10, and CXCL9 and subsequently decreasing the recruitment of effector CD8 T cells, thereby exerting resistance to checkpoint immunotherapy. Interestingly, ssGSEA results and correlation analysis show that signaling pathways related to tumor invasion and metastasis, such as p53 signaling pathway, EMT2, EMT3, and TGF-beta signaling pathway, were mainly involved in high-risk groups, which are recognized as immunosuppressive and play a key role in tumorigenesis [26]. However, CD8 T effector was significantly involved in low-risk group, which indicates that the low-risk group was immune activation and maybe respond better to immunotherapy.

The immunogenicity of the tumor is the basis of initiating antitumor immune response, and higher frequency of somatic mutations may lead to more neoantigens produced by tumor cells and improve the immune killing ability of T cells to tumor cells [27]. TMB, defined as the total number of somatic gene non-synonymous mutations, is considered an effective indicator for tumor immunotherapy [28]. In our research, the IRL score was significantly negatively correlated with TMB, which hints that the IRL classifier maybe predicts immunotherapeutic efficiency in LSCC. According to TIDE algorithm, there were more immunotherapy responders in the IRL classifier low-risk groups, and the IRL classifier was robustly negatively linked to the immunotherapeutic response. Hence, IRL classifier was proved to be efficient for the immunotherapy response prediction in LSCC cohort. All of these supported that IRL classifier was a potent tool for determining the immunotherapy sensitivity for LSCC patients.

While significant, our research inevitably has limitations. First, we merely extract retrospectively imperfect data (TCGA and GEO datasets), analyzing them through biological algorithm approaches. Therefore, our results still need to be externally validated in large sample sizes multicenter prospective cohorts. Second, while bioinformatics tools are helpful in exploiting the discovery of novel biomarkers for diagnosis, treatment, and prognosis, in vitro and vivo experiments in LSCC are also of importance to further elucidate the molecular mechanisms of hub IRLs.

Finally, in fact, patients with LSCC did not receive corresponding ICIs treatment, and the response to immunotherapy was computed using cutting-edge bioinformatics technologies. Though the IRL classifier can stratify LSCC patients with different immune responses, external data validation is lacking. Hence, multicenter large-scale studies are needed to evaluate its usefulness in clinical trials and strengthen its clinical evidence.

## Conclusion

Based on BARX1-DT, KLHL7-DT, and LINC02154, the IRL classifier was established, which can be utilized to predict the prognosis, immune infiltration, and immunotherapeutic efficiency in patients with LSCC and might facilitate individualized counseling for immunotherapy.

## Supplementary Information

**Additional file 1: Fig. S1.** The survival curves of individual IRL (BARX1-DT, KLHL7-DT, and LINC02154) for prediction of OS in patients with LSCC. A: BARX1-DT in TCGA database. B: BARX1-DT in TCGA database. C: LINC02154 in TCGA database. A: BARX1-DT in TCGA database. D: BARX1-DT in GEO database. E: BARX1-DT in GEO database. F: LINC02154 in GEO database.

**Additional file 2.** **Supplementary material 1.**

**Additional file 3.** **Supplementary material 2.**

**Additional file 4.** **Supplementary material 3.**

## Data Availability

The data that support the findings of this study are provided in supplementary materials and are also made available in the TCGA (https://gdc.cancer.gov/) and GEO (https://www.ncbi.nlm.nih.gov/geo/).

## Abbreviations

IRLs: Immune-related long noncoding RNAs
lncRNAs: Long noncoding RNAs
LSCC: Laryngeal squamous cell carcinoma
RNA-seq: RNA sequencing
TCGA: The Cancer Genome Atlas
LASSO: Least absolute shrinkage and selection operator
GEO: Gene Expression Omnibus
RF: Random forest
ROC: Receiver operating characteristic
RFS: Recurrence-free survival
AUC: Area under curve
DCA: Decision curve analysis
IRG: Immune-related genes
ssGSEA: Single-sample gene set enrichment analysis
TME: Tumor microenvironment
TMB: Tumor mutation burden
MSI: Microsatellite instability
TIDE: Tumor immune dysfunction and exclusion
OS: Overall survival
ICIs: Immune checkpoint inhibitors
DEIRLs: Differential expression immune-related long noncoding RNAs
FDR: False discovery rate
APC: Antigen-presenting cell
GSVA: Gene set variation analysis
MSigDB: Molecular signatures database
