# Supplementary material for: Construction and validation of immune-related LncRNAs classifier to predict prognosis and immunotherapy response in laryngeal squamous cell carcinoma
Source: World J Surg Oncol. 2022 May 24;20:164. doi: 10.1186/s12957-022-02608-z (PMC9128282; doi:10.1186/s12957-022-02608-z)
Supplement: Supplementary file 2 — Additional file 2. Supplementary material 1. [file 12957_2022_2608_MOESM2_ESM.doc]

**数据合并**

setwd("F:\\科研论文\\科研学术\\生信研究\\LSCC\\IRLs\\数据分析\\TCGA")

inputfile1="ImmuneGene.txt" #生存时间数据

inputfile2="RSymbol.txt" #差异基因表达数据

time_data<-read.table(inputfile1,header = T,sep = "\t",check.names = F)

geneEXP<-read.table(inputfile2,header = T,sep = "\t",check.names = F)

head(time_data)

head(geneEXP)

merger_data<-merge(time_data,geneEXP,by="id")

write.table(merger_data,"ImmuneGeneExp.txt",sep = "\t",row.names = F,quote = F)

setwd("F:\\科研论文\\科研学术\\生信研究\\LSCC\\IRLs\\数据分析\\TCGA")

inputfile1="LncRNA.txt" #生存时间数据

inputfile2="RSymbol.txt" #差异基因表达数据

time_data<-read.table(inputfile1,header = T,sep = "\t",check.names = F)

geneEXP<-read.table(inputfile2,header = T,sep = "\t",check.names = F)

head(time_data)

head(geneEXP)

merger_data<-merge(time_data,geneEXP,by="id")

write.table(merger_data,"LncRNAExp.txt",sep = "\t",row.names = F,quote = F)

**Immune LncRNA（Cor）**

library(limma)

setwd("F:\\科研论文\\科研学术\\生信研究\\LSCC\\IRLs\\数据分析\\TCGA")

corFilter=0.4 #相关系数过滤标准

pvalueFilter=0.05 #p值过滤标准

#读取lncRNA表达文件,并对数据进行处理

rt = read.table("LncRNAExp.txt",header=T,sep="\t",check.names=F)

rt=as.matrix(rt)

rownames(rt)=rt[,1]

exp=rt[,2:ncol(rt)]

dimnames=list(rownames(exp),colnames(exp))

lncRNA=matrix(as.numeric(as.matrix(exp)),nrow=nrow(exp),dimnames=dimnames)

lncRNA=avereps(lncRNA)

lncRNA=lncRNA[rowMeans(lncRNA)>0.5,]

group=sapply(strsplit(colnames(lncRNA),"\\-"),"[",4)

group=sapply(strsplit(group,""),"[",1)

group=gsub("2","1",group)

lncRNA=lncRNA[,group==0]

#读取免疫基因表达文件,并对数据进行处理

rt = read.table("ImmuneGeneExp.txt",header=T,sep="\t",check.names=F)

rt=as.matrix(rt)

rownames(rt)=rt[,1]

exp=rt[,2:ncol(rt)]

dimnames=list(rownames(exp),colnames(exp))

immuneGene=matrix(as.numeric(as.matrix(exp)),nrow=nrow(exp),dimnames=dimnames)

immuneGene=avereps(immuneGene)

immuneGene=immuneGene[rowMeans(immuneGene)>0.5,]

group=sapply(strsplit(colnames(immuneGene),"\\-"),"[",4)

group=sapply(strsplit(group,""),"[",1)

group=gsub("2","1",group)

immuneGene=immuneGene[,group==0]

#相关性检验

outTab=data.frame()

for(i in row.names(lncRNA)){

if(sd(lncRNA[i,])>0.5){

for(j in row.names(immuneGene)){

x=as.numeric(lncRNA[i,])

y=as.numeric(immuneGene[j,])

corT=cor.test(x,y)

cor=corT$estimate

pvalue=corT$p.value

if((cor>corFilter) & (pvalue<pvalueFilter)){

outTab=rbind(outTab,cbind(immuneGene=j,lncRNA=i,cor,pvalue,Regulation="postive"))

}

if((cor< -corFilter) & (pvalue<pvalueFilter)){

outTab=rbind(outTab,cbind(immuneGene=j,lncRNA=i,cor,pvalue,Regulation="negative"))

}

}

}

}

write.table(file="CorResult.txt",outTab,sep="\t",quote=F) #输出相关性结果

immuneLncRNA=unique(outTab[,"lncRNA"])

immuneLncRNAexp=lncRNA[immuneLncRNA,]

immuneLncRNAexp=rbind(ID=colnames(immuneLncRNAexp),immuneLncRNAexp)

write.table(immuneLncRNAexp,file="ImmuneLncRNAexp.txt",sep="\t",quote=F,col.names=F)

**ImmuneLncRNAexp**

setwd("F:\\科研论文\\科研学术\\生信研究\\LSCC\\IRLs\\数据分析\\差异分析")

inputfile1="LncRNA.txt" #生存时间数据

inputfile2="LncRNAExp.txt" #差异基因表达数据

time_data<-read.table(inputfile1,header = T,sep = "\t",check.names = F)

geneEXP<-read.table(inputfile2,header = T,sep = "\t",check.names = F)

head(time_data)

head(geneEXP)

merger_data<-merge(time_data,geneEXP,by="id")

write.table(merger_data,"ImmuneLncRNAexp.txt",sep = "\t",row.names = F,quote = F)

**DEIRL**

setwd("F:\\科研论文\\科研学术\\生信研究\\LSCC\\IRLs\\数据分析\\差异分析")

inputfile1="DEIRL.txt" #生存时间数据

inputfile2="LncRNAExp.txt" #差异基因表达数据

time_data<-read.table(inputfile1,header = T,sep = "\t",check.names = F)

geneEXP<-read.table(inputfile2,header = T,sep = "\t",check.names = F)

head(time_data)

head(geneEXP)

merger_data<-merge(time_data,geneEXP,by="id")

write.table(merger_data,"DEIRLexp.txt",sep = "\t",row.names = F,quote = F)

**GEO DEIRL**

setwd("F:\\科研论文\\科研学术\\生信研究\\LSCC\\IRLs\\数据分析\\GEO\\GSE25727")

inputfile1="DEIRL.txt" #生存时间数据

inputfile2="geneMatrix.txt" #差异基因表达数据

time_data<-read.table(inputfile1,header = T,sep = "\t",check.names = F)

geneEXP<-read.table(inputfile2,header = T,sep = "\t",check.names = F)

head(time_data)

head(geneEXP)

merger_data<-merge(time_data,geneEXP,by="id")

write.table(merger_data,"DEIRLexp.txt",sep = "\t",row.names = F,quote = F)

setwd("F:\\科研论文\\科研学术\\生信研究\\LSCC\\IRLs\\数据分析\\GEO\\GSE27020")

inputfile1="DEIRL.txt" #生存时间数据

inputfile2="geneMatrix.txt" #差异基因表达数据

time_data<-read.table(inputfile1,header = T,sep = "\t",check.names = F)

geneEXP<-read.table(inputfile2,header = T,sep = "\t",check.names = F)

head(time_data)

head(geneEXP)

merger_data<-merge(time_data,geneEXP,by="id")

write.table(merger_data,"DEIRLexp.txt",sep = "\t",row.names = F,quote = F)

setwd("F:\\科研论文\\科研学术\\生信研究\\LSCC\\IRLs\\数据分析\\GEO\\GSE65858")

inputfile1="DEIRL.txt" #生存时间数据

inputfile2="geneMatrix.txt" #差异基因表达数据

time_data<-read.table(inputfile1,header = T,sep = "\t",check.names = F)

geneEXP<-read.table(inputfile2,header = T,sep = "\t",check.names = F)

head(time_data)

head(geneEXP)

merger_data<-merge(time_data,geneEXP,by="id")

write.table(merger_data,"DEIRLexp.txt",sep = "\t",row.names = F,quote = F)

**Cox回归**

setwd("F:\\科研论文\\科研学术\\生信研究\\LSCC\\IRLs\\数据分析\\Cox")

inputfile1="Clinical.txt" #生存时间数据

inputfile2="DEIRLex.txt" #差异基因表达数据

time_data<-read.table(inputfile1,header = T,sep = "\t",check.names = F)

geneEXP<-read.table(inputfile2,header = T,sep = "\t",check.names = F)

head(time_data)

head(geneEXP)

merger_data<-merge(time_data,geneEXP,by="id")

write.table(merger_data,"Cox.txt",sep = "\t",row.names = F,quote = F)

library(survival)

setwd("F:\\科研论文\\科研学术\\生信研究\\LSCC\\IRLs\\数据分析\\Cox")

lncRNA<-read.table("Cox.txt",header=T,sep="\t",row.names = 1,check.names = F)

coxR=data.frame()

coxf<-function(x){

fmla1 <- as.formula(Surv(Time,OS)~lncRNA[,x])

mycox <- coxph(fmla1,data=lncRNA)

}

for(a in colnames(lncRNA[,3:ncol(lncRNA)])){

mycox=coxf(a)

coxResult = summary(mycox)

coxR=rbind(coxR,cbind(lncRNAname=a,HR=coxResult$coefficients[,"exp(coef)"],

P=coxResult$coefficients[,"Pr(>|z|)"]))

}

write.table(coxR,"CoxResult.txt",sep="\t",row.names=F,quote=F)

**变量选择**

setwd("F:\\科研论文\\科研学术\\生信研究\\LSCC\\IRLs\\数据分析\\变量选择")

inputfile1="IRL.txt" #生存时间数据

inputfile2="MDEIRLexp.txt" #差异基因表达数据

time_data<-read.table(inputfile1,header = T,sep = "\t",check.names = F)

geneEXP<-read.table(inputfile2,header = T,sep = "\t",check.names = F)

head(time_data)

head(geneEXP)

merger_data<-merge(time_data,geneEXP,by="id")

write.table(merger_data,"MLA.txt",sep = "\t",row.names = F,quote = F)

**Lasso回归-Cox Regression**

setwd("D:\\科研论文\\科研学术\\生信研究\\LSCC\\IRLs\\数据分析\\变量选择\\LASSO")

library(glmnet)

library(survival)

lncRNA<-read.table("LASSO.txt",header=T,sep="\t",row.names = 1,check.names = F,stringsAsFactors = F)

v1<-as.matrix(lncRNA[,c(3:ncol(lncRNA))])

v2 <- as.matrix(Surv(lncRNA$Time,lncRNA$OS))

myfit <- glmnet(v1, v2, family = "cox")

plot(myfit, xvar = "lambda", label =FALSE)

myfit1 <- cv.glmnet(v1, v2, family="cox")

plot(myfit1)

abline(v=log(c(myfit1$lambda.min,myfit1$lambda.1se)),lty="dashed")

myfit1$lambda.min

myfit1$lambda.1se

coe <- coef(myfit, s = myfit1$lambda.min)

act_index <- which(coe != 0)

act_coe <- coe[act_index]

row.names(coe)[act_index]

myfit1$lambda.min

myfit1$lambda.1se

coef(myfit1,s="lambda.min")

**RF**

library(randomForest) #random forests

library(caret) #

setwd("D:\\科研论文\\科研学术\\生信研究\\LSCC\\IRLs\\数据分析\\变量选择\\RF")

data=read.table("RF.txt",sep="\t",header=T,check.names=F, row.names = 1)

rf.pros <- randomForest(OS ~ ., data = data)

rf.pros

plot(rf.pros)

which.min(rf.pros$mse)

rf.pros.2 <- randomForest(OS~ ., data =data, ntree = 484)

rf.pros.2

varImpPlot(rf.pros.2, scale = TRUE,

main = "Variable Importance Plot - IRL")

importance(rf.pros.2)

**VEEN**

library("ggvenn")

setwd("D:\\科研论文\\科研学术\\生信研究\\LSCC\\IRLs\\数据分析\\变量选择\\Venn")

data1<-read.delim("LASSO.txt",header = T,stringsAsFactors = F) #读入数据

data2<-read.delim("RF.txt",header = T,stringsAsFactors = F) #读入数据

data3<-read.delim("PRIRL.txt",header = T,stringsAsFactors = F) #读入数据

x<-list(LASSO=data1$A,RF=data2$B,PRIRL=data3$C)

ggvenn(x,c("LASSO","RF","PRIRL"),

show_percentage = F,

stroke_color="white",#边界线的颜色

fill_color = c("#ffb2b2", "#b2e7cd", "#b2d4ec"),

set_name_color = c("#ff0000", "#4a9b83", "#1d6295"), text_size = 5)#调节字体大小

**模型构建**

**TCGA OS**

library(survival)

setwd("D:\\科研论文\\科研学术\\生信研究\\LSCC\\IRLs\\数据分析\\预后模型\\OS\\TCGA")

lncRNA<-read.table("MCox.txt",header=T,sep="\t",row.names = 1,check.names = F,stringsAsFactors = F)

mycox <- coxph(Surv(Time,OS ==1)~ .,data=lncRNA)

summary(mycox)

riskscore<-predict(mycox,type="risk",newdata=lncRNA)

risklevel<-as.factor(ifelse(riskscore>median(riskscore),"High","Low"))

write.table(cbind(id=rownames(cbind(lncRNA[,1:5],riskscore,risklevel)),cbind(lncRNA[,1:5],riskscore,risklevel)),"RiskScore.txt",sep="\t",quote=F,row.names=F)

**ROC曲线**

library(survival)

library(timeROC)

setwd("D:\\科研论文\\科研学术\\生信研究\\LSCC\\IRLs\\数据分析\\预后模型\\OS\\TCGA")

risk=read.table("RiskScore.txt",header=T,sep="\t")

predict_3_year<- 36

predict_5_year<- 60

ROC<-timeROC(T=risk$Time,delta=risk$OS,

marker=risk$riskscore,cause=1,

weighting="marginal",

times=c(predict_3_year,predict_5_year),ROC=TRUE)

plot(ROC,time=predict_3_year,title=FALSE, lwd=2)

plot(ROC,time=predict_5_year, col="blue",add=TRUE,title=FALSE,lwd=2)

legend("bottomright",

c(paste("AUC of 3 year OS: ",round(ROC$AUC[1],3)),

paste("AUC of 5 year OS: ",round(ROC$AUC[2],3))),col=c("red","blue"),lwd=2)

abline(0,1,lty=3,lwd=1.5,col=c(rgb(0,0,0,maxColorValue=255)))

**生存函数图**

library(survival)

setwd("D:\\科研论文\\科研学术\\生信研究\\LSCC\\IRLs\\数据分析\\预后模型\\OS\\TCGA")

Risk=read.table("RiskScore.txt",header=T,sep="\t")

diff=survdiff(Surv(Time, OS) ~risklevel,data = Risk)

pValue=1-pchisq(diff$chisq,df=1)

pValue=signif(pValue,4)

pValue=format(pValue, scientific = TRUE)

fit <- survfit(Surv(Time, OS) ~ risklevel, data =Risk)

plot(fit, lwd=2,col=c("red","blue"),

xlab="Time (months)",

ylab="Overall survival",

mark.time=T)

text(30,0.1,paste("***P***value:", pValue,sep=""),col="black")

legend("topright",

c("High risk", "Low risk"),

lwd=2,

col=c("red","blue"))

**风险图**

setwd("D:\\科研论文\\科研学术\\生信研究\\LSCC\\IRLs\\数据分析\\预后模型\\OS\\TCGA")

rt=read.table("RiskScore.txt",header=T,sep="\t", row.names=1,check.names=F)

rt=rt[order(rt$riskscore),]

riskClass=rt[,"risklevel"]

lowLength=length(riskClass[riskClass=="Low"])

highLength=length(riskClass[riskClass=="High"])

line=rt[,"riskscore"]

line[line>10]=10

plot(line,type="p",pch=20,

xlab="Patients (increasing risk socre)",

ylab="Risk score",

col=c(rep("green", lowLength),

rep("red", highLength)))

abline(h=median(rt$riskscore),v=lowLength,lty=2)

**生存图**

setwd("D:\\科研论文\\科研学术\\生信研究\\LSCC\\IRLs\\数据分析\\预后模型\\OS\\TCGA")

rt=read.table("RiskScore.txt",header=T,sep="\t", row.names=1,check.names=F)

rt=rt[order(rt$riskscore),]

riskClass=rt[,"risklevel"]

lowLength=length(riskClass[riskClass=="Low"])

highLength=length(riskClass[riskClass=="High"])

color=as.vector(rt$OS)

color[color==1]="red"

color[color==0]="green"

plot(rt$Time,

pch=19,

xlab="Patients (increasing risk socre)",

ylab="Overall survival",

col=color)

legend("topright",

c("Death", "Survival"),

pch=19,

col=c("red","green"))

abline(v=lowLength,lty=2)

**TCGA RFS**

setwd("D:\\科研论文\\科研学术\\生信研究\\LSCC\\IRLs\\数据分析\\预后模型\\RFS\\TCGA")

inputfile1="Clinical.txt" #生存时间数据

inputfile2="MCox.txt" #差异基因表达数据

time_data<-read.table(inputfile1,header = T,sep = "\t",check.names = F)

geneEXP<-read.table(inputfile2,header = T,sep = "\t",check.names = F)

head(time_data)

head(geneEXP)

merger_data<-merge(time_data,geneEXP,by="id")

write.table(merger_data,"RMCox.txt",sep = "\t",row.names = F,quote = F)

library(survival)

setwd("D:\\科研论文\\科研学术\\生信研究\\LSCC\\IRLs\\数据分析\\预后模型\\RFS\\TCGA")

lncRNA<-read.table("RMCox.txt",header=T,sep="\t",row.names = 1,check.names = F,stringsAsFactors = F)

mycox <- coxph(Surv(Time,RFS ==1)~ .,data=lncRNA)

summary(mycox)

riskscore<-predict(mycox,type="risk",newdata=lncRNA)

risklevel<-as.factor(ifelse(riskscore>median(riskscore),"High","Low"))

write.table(cbind(id=rownames(cbind(lncRNA[,1:5],riskscore,risklevel)),cbind(lncRNA[,1:5],riskscore,risklevel)),"RiskScore.txt",sep="\t",quote=F,row.names=F)

**ROC曲线**

library(survival)

library(timeROC)

setwd("D:\\科研论文\\科研学术\\生信研究\\LSCC\\IRLs\\数据分析\\预后模型\\RFS\\TCGA")

risk=read.table("RiskScore.txt",header=T,sep="\t")

predict_3_year<- 36

predict_5_year<- 60

ROC<-timeROC(T=risk$Time,delta=risk$RFS,

marker=risk$riskscore,cause=1,

weighting="marginal",

times=c(predict_3_year,predict_5_year),ROC=TRUE)

plot(ROC,time=predict_3_year,title=FALSE, lwd=2)

plot(ROC,time=predict_5_year, col="blue",add=TRUE,title=FALSE,lwd=2)

legend("bottomright",

c(paste("AUC of 3 year RFS: ",round(ROC$AUC[1],3)),

paste("AUC of 5 year RFS: ",round(ROC$AUC[2],3))),col=c("red","blue"),lwd=2)

abline(0,1,lty=3,lwd=1.5,col=c(rgb(0,0,0,maxColorValue=255)))

**生存函数图**

library(survival)

setwd("D:\\科研论文\\科研学术\\生信研究\\LSCC\\IRLs\\数据分析\\预后模型\\RFS\\TCGA")

Risk=read.table("RiskScore.txt",header=T,sep="\t")

diff=survdiff(Surv(Time, RFS) ~risklevel,data = Risk)

pValue=1-pchisq(diff$chisq,df=1)

pValue=signif(pValue,4)

pValue=format(pValue, scientific = TRUE)

fit <- survfit(Surv(Time, RFS) ~ risklevel, data =Risk)

plot(fit, lwd=2,col=c("red","blue"),

xlab="Time (months)",

ylab="Recurrence-free survival",

mark.time=T)

text(30,0.1,paste("***P***value:", pValue,sep=""),col="black")

legend("topright",

c("High risk", "Low risk"),

lwd=2,

col=c("red","blue"))

**GSE65858 OS**

setwd("D:\\科研论文\\科研学术\\生信研究\\LSCC\\IRLs\\数据分析\\预后模型\\OS\\GSE65858")

inputfile1="Cli.txt" #生存时间数据

inputfile2="DEIRLexp.txt" #差异基因表达数据

time_data<-read.table(inputfile1,header = T,sep = "\t",check.names = F)

geneEXP<-read.table(inputfile2,header = T,sep = "\t",check.names = F)

head(time_data)

head(geneEXP)

merger_data<-merge(time_data,geneEXP,by="id")

write.table(merger_data,"Cox.txt",sep = "\t",row.names = F,quote = F)

**转置**

setwd("D:\\科研论文\\科研学术\\生信研究\\LSCC\\IRLs\\数据分析\\预后模型\\OS\\GSE65858")

inputfile1="geneMatrix.txt"

time_data<-read.table(inputfile1,header = T,sep = "\t",check.names = F)

rt<-t(time_data)

write.table(rt,"RgeneMatrix.txt",sep = "\t",row.names = F,quote = F)

setwd("D:\\科研论文\\科研学术\\生信研究\\LSCC\\IRLs\\数据分析\\预后模型\\OS\\GSE65858")

inputfile1="id.txt" #生存时间数据

inputfile2="RgeneMatrix.txt" #差异基因表达数据

time_data<-read.table(inputfile1,header = T,sep = "\t",check.names = F)

geneEXP<-read.table(inputfile2,header = T,sep = "\t",check.names = F)

head(time_data)

head(geneEXP)

merger_data<-merge(time_data,geneEXP,by="id")

write.table(merger_data,"LSCC.txt",sep = "\t",row.names = F,quote = F)

**转置**

setwd("D:\\科研论文\\科研学术\\生信研究\\LSCC\\IRLs\\数据分析\\预后模型\\OS\\GSE65858")

inputfile1="LSCC.txt"

time_data<-read.table(inputfile1,header = T,sep = "\t",check.names = F)

rt<-t(time_data)

write.table(rt,"LSCCExp.txt",sep = "\t",row.names = F,quote = F)

library(survival)

setwd("D:\\科研论文\\科研学术\\生信研究\\LSCC\\IRLs\\数据分析\\预后模型\\OS\\GSE65858")

lncRNA<-read.table("Cox.txt",header=T,sep="\t",row.names = 1,check.names = F)

coxR=data.frame()

coxf<-function(x){

fmla1 <- as.formula(Surv(Time,OS)~lncRNA[,x])

mycox <- coxph(fmla1,data=lncRNA)

}

for(a in colnames(lncRNA[,5:ncol(lncRNA)])){

mycox=coxf(a)

coxResult = summary(mycox)

coxR=rbind(coxR,cbind(lncRNAname=a,HR=coxResult$coefficients[,"exp(coef)"],

P=coxResult$coefficients[,"Pr(>|z|)"]))

}

write.table(coxR,"CoxResult.txt",sep="\t",row.names=F,quote=F)

library(survival)

setwd("D:\\科研论文\\科研学术\\生信研究\\LSCC\\IRLs\\数据分析\\预后模型\\OS\\GSE65858")

lncRNA<-read.table("OMCox.txt",header=T,sep="\t",row.names = 1,check.names = F,stringsAsFactors = F)

mycox <- coxph(Surv(Time,OS ==1)~., data=lncRNA)

summary(mycox)

riskscore<-predict(mycox,type="risk",newdata=lncRNA)

risklevel<-as.factor(ifelse(riskscore>median(riskscore),"High","Low"))

write.table(cbind(id=rownames(cbind(lncRNA[,1:5],riskscore,risklevel)),cbind(lncRNA[,1:5],riskscore,risklevel)),"RiskScore.txt",sep="\t",quote=F,row.names=F)

**ROC曲线**

library(survival)

library(timeROC)

setwd("D:\\科研论文\\科研学术\\生信研究\\LSCC\\IRLs\\数据分析\\预后模型\\OS\\GSE65858")

risk=read.table("RiskScore.txt",header=T,sep="\t")

predict_3_year<- 36

predict_5_year<- 60

ROC<-timeROC(T=risk$Time,delta=risk$OS,

marker=risk$riskscore,cause=1,

weighting="marginal",

times=c(predict_3_year,predict_5_year),ROC=TRUE)

plot(ROC,time=predict_3_year,title=FALSE, lwd=2)

plot(ROC,time=predict_5_year, col="blue",add=TRUE,title=FALSE,lwd=2)

legend("bottomright",

c(paste("AUC of 3 year OS: ",round(ROC$AUC[1],3)),

paste("AUC of 5 year OS: ",round(ROC$AUC[2],3))),col=c("red","blue"),lwd=2)

abline(0,1,lty=3,lwd=1.5,col=c(rgb(0,0,0,maxColorValue=255)))

**生存函数图**

library(survival)

setwd("D:\\科研论文\\科研学术\\生信研究\\LSCC\\IRLs\\数据分析\\预后模型\\OS\\GSE65858")

Risk=read.table("RiskScore.txt",header=T,sep="\t")

diff=survdiff(Surv(Time, OS) ~risklevel,data = Risk)

pValue=1-pchisq(diff$chisq,df=1)

pValue=signif(pValue,4)

pValue=format(pValue, scientific = TRUE)

fit <- survfit(Surv(Time, OS) ~ risklevel, data =Risk)

plot(fit, lwd=2,col=c("red","blue"),

xlab="Time (months)",

ylab="Overall survival",

mark.time=T)

text(30,0.1,paste("***P***value:", pValue,sep=""),col="black")

legend("topright",

c("High risk", "Low risk"),

lwd=2,

col=c("red","blue"))

**风险图**

setwd("D:\\科研论文\\科研学术\\生信研究\\LSCC\\IRLs\\数据分析\\预后模型\\OS\\GSE65858")

rt=read.table("RiskScore.txt",header=T,sep="\t", row.names=1,check.names=F)

rt=rt[order(rt$riskscore),]

riskClass=rt[,"risklevel"]

lowLength=length(riskClass[riskClass=="Low"])

highLength=length(riskClass[riskClass=="High"])

line=rt[,"riskscore"]

line[line>10]=10

plot(line,type="p",pch=20,

xlab="Patients (increasing risk socre)",

ylab="Risk score",

col=c(rep("green", lowLength),

rep("red", highLength)))

abline(h=median(rt$riskscore),v=lowLength,lty=2)

**生存图**

setwd("D:\\科研论文\\科研学术\\生信研究\\LSCC\\IRLs\\数据分析\\预后模型\\OS\\GSE65858")

rt=read.table("RiskScore.txt",header=T,sep="\t", row.names=1,check.names=F)

rt=rt[order(rt$riskscore),]

riskClass=rt[,"risklevel"]

lowLength=length(riskClass[riskClass=="Low"])

highLength=length(riskClass[riskClass=="High"])

color=as.vector(rt$OS)

color[color==1]="red"

color[color==0]="green"

plot(rt$Time,

pch=19,

xlab="Patients (increasing risk socre)",

ylab="Overall survival",

col=color)

legend("topright",

c("Death", "Survival"),

pch=19,

col=c("red","green"))

abline(v=lowLength,lty=2)

**GSE65858 RFS**

**ROC曲线**

library(survival)

library(timeROC)

setwd("D:\\科研论文\\科研学术\\生信研究\\LSCC\\IRLs\\数据分析\\预后模型\\RFS\\GSE65858")

risk=read.table("RiskScore.txt",header=T,sep="\t")

predict_3_year<- 36

predict_5_year<- 60

ROC<-timeROC(T=risk$Time,delta=risk$RFS,

marker=risk$riskscore,cause=1,

weighting="marginal",

times=c(predict_3_year,predict_5_year),ROC=TRUE)

plot(ROC,time=predict_3_year,title=FALSE, lwd=2)

plot(ROC,time=predict_5_year, col="blue",add=TRUE,title=FALSE,lwd=2)

legend("bottomright",

c(paste("AUC of 3 year RFS: ",round(ROC$AUC[1],3)),

paste("AUC of 5 year RFS: ",round(ROC$AUC[2],3))),col=c("red","blue"),lwd=2)

abline(0,1,lty=3,lwd=1.5,col=c(rgb(0,0,0,maxColorValue=255)))

**生存函数图**

library(survival)

setwd("D:\\科研论文\\科研学术\\生信研究\\LSCC\\IRLs\\数据分析\\预后模型\\RFS\\GSE65858")

Risk=read.table("RiskScore.txt",header=T,sep="\t")

diff=survdiff(Surv(Time, RFS) ~risklevel,data = Risk)

pValue=1-pchisq(diff$chisq,df=1)

pValue=signif(pValue,4)

pValue=format(pValue, scientific = TRUE)

fit <- survfit(Surv(Time, RFS) ~ risklevel, data =Risk)

plot(fit, lwd=2,col=c("red","blue"),

xlab="Time (months)",

ylab="Recurrence-free survival",

mark.time=T)

text(30,0.1,paste("***P***value:", pValue,sep=""),col="black")

legend("topright",

c("High risk", "Low risk"),

lwd=2,

col=c("red","blue"))

**GSE25727 RFS**

library(survival)

setwd("D:\\科研论文\\科研学术\\生信研究\\LSCC\\IRLs\\数据分析\\预后模型\\RFS\\GSE25727")

lncRNA<-read.table("Cox.txt",header=T,sep="\t",row.names = 1,check.names = F)

coxR=data.frame()

coxf<-function(x){

fmla1 <- as.formula(Surv(Time,RFS)~lncRNA[,x])

mycox <- coxph(fmla1,data=lncRNA)

}

for(a in colnames(lncRNA[,5:ncol(lncRNA)])){

mycox=coxf(a)

coxResult = summary(mycox)

coxR=rbind(coxR,cbind(lncRNAname=a,HR=coxResult$coefficients[,"exp(coef)"],

P=coxResult$coefficients[,"Pr(>|z|)"]))

}

write.table(coxR,"CoxResult.txt",sep="\t",row.names=F,quote=F)

library(survival)

setwd("D:\\科研论文\\科研学术\\生信研究\\LSCC\\IRLs\\数据分析\\预后模型\\RFS\\GSE25727")

lncRNA<-read.table("MCox.txt",header=T,sep="\t",row.names = 1,check.names = F,stringsAsFactors = F)

mycox <- coxph(Surv(Time,RFS ==1)~., data=lncRNA)

summary(mycox)

riskscore<-predict(mycox,type="risk",newdata=lncRNA)

risklevel<-as.factor(ifelse(riskscore>median(riskscore),"High","Low"))

write.table(cbind(id=rownames(cbind(lncRNA[,1:5],riskscore,risklevel)),cbind(lncRNA[,1:5],riskscore,risklevel)),"RiskScore.txt",sep="\t",quote=F,row.names=F)

**ROC曲线**

library(survival)

library(timeROC)

setwd("D:\\科研论文\\科研学术\\生信研究\\LSCC\\IRLs\\数据分析\\预后模型\\RFS\\GSE25727")

risk=read.table("RiskScore.txt",header=T,sep="\t")

predict_3_year<- 36

predict_5_year<- 60

ROC<-timeROC(T=risk$Time,delta=risk$RFS,

marker=risk$riskscore,cause=1,

weighting="marginal",

times=c(predict_3_year,predict_5_year),ROC=TRUE)

plot(ROC,time=predict_3_year,title=FALSE, lwd=2)

plot(ROC,time=predict_5_year, col="blue",add=TRUE,title=FALSE,lwd=2)

legend("bottomright",

c(paste("AUC of 3 year RFS: ",round(ROC$AUC[1],3)),

paste("AUC of 5 year RFS: ",round(ROC$AUC[2],3))),col=c("red","blue"),lwd=2)

abline(0,1,lty=3,lwd=1.5,col=c(rgb(0,0,0,maxColorValue=255)))

**生存函数图**

library(survival)

setwd("D:\\科研论文\\科研学术\\生信研究\\LSCC\\IRLs\\数据分析\\预后模型\\RFS\\GSE65858")

Risk=read.table("RiskScore.txt",header=T,sep="\t")

diff=survdiff(Surv(Time, RFS) ~risklevel,data = Risk)

pValue=1-pchisq(diff$chisq,df=1)

pValue=signif(pValue,4)

pValue=format(pValue, scientific = TRUE)

fit <- survfit(Surv(Time, RFS) ~ risklevel, data =Risk)

plot(fit, lwd=2,col=c("red","blue"),

xlab="Time (months)",

ylab="Recurrence-free survival",

mark.time=T)

text(30,0.1,paste("***P***value:", pValue,sep=""),col="black")

legend("topright",

c("High risk", "Low risk"),

lwd=2,

col=c("red","blue"))

**GSE27020 RFS**

library(survival)

setwd("D:\\科研论文\\科研学术\\生信研究\\LSCC\\IRLs\\数据分析\\预后模型\\RFS\\GSE27020")

lncRNA<-read.table("Cox.txt",header=T,sep="\t",row.names = 1,check.names = F)

coxR=data.frame()

coxf<-function(x){

fmla1 <- as.formula(Surv(Time,RFS)~lncRNA[,x])

mycox <- coxph(fmla1,data=lncRNA)

}

for(a in colnames(lncRNA[,5:ncol(lncRNA)])){

mycox=coxf(a)

coxResult = summary(mycox)

coxR=rbind(coxR,cbind(lncRNAname=a,HR=coxResult$coefficients[,"exp(coef)"],

P=coxResult$coefficients[,"Pr(>|z|)"]))

}

write.table(coxR,"CoxResult.txt",sep="\t",row.names=F,quote=F)

library(survival)

setwd("D:\\科研论文\\科研学术\\生信研究\\LSCC\\IRLs\\数据分析\\预后模型\\RFS\\GSE27020")

lncRNA<-read.table("MCox.txt",header=T,sep="\t",row.names = 1,check.names = F,stringsAsFactors = F)

mycox <- coxph(Surv(Time,RFS ==1)~., data=lncRNA)

summary(mycox)

riskscore<-predict(mycox,type="risk",newdata=lncRNA)

risklevel<-as.factor(ifelse(riskscore>median(riskscore),"High","Low"))

write.table(cbind(id=rownames(cbind(lncRNA[,1:5],riskscore,risklevel)),cbind(lncRNA[,1:5],riskscore,risklevel)),"RiskScore.txt",sep="\t",quote=F,row.names=F)

**ROC曲线**

library(survival)

library(timeROC)

setwd("D:\\科研论文\\科研学术\\生信研究\\LSCC\\IRLs\\数据分析\\预后模型\\RFS\\GSE27020")

risk=read.table("RiskScore.txt",header=T,sep="\t")

predict_3_year<- 36

predict_5_year<- 60

ROC<-timeROC(T=risk$Time,delta=risk$RFS,

marker=risk$riskscore,cause=1,

weighting="marginal",

times=c(predict_3_year,predict_5_year),ROC=TRUE)

plot(ROC,time=predict_3_year,title=FALSE, lwd=2)

plot(ROC,time=predict_5_year, col="blue",add=TRUE,title=FALSE,lwd=2)

legend("bottomright",

c(paste("AUC of 3 year RFS: ",round(ROC$AUC[1],3)),

paste("AUC of 5 year RFS: ",round(ROC$AUC[2],3))),col=c("red","blue"),lwd=2)

abline(0,1,lty=3,lwd=1.5,col=c(rgb(0,0,0,maxColorValue=255)))

**生存函数图**

library(survival)

setwd("D:\\科研论文\\科研学术\\生信研究\\LSCC\\IRLs\\数据分析\\预后模型\\RFS\\GSE27020")

Risk=read.table("RiskScore.txt",header=T,sep="\t")

diff=survdiff(Surv(Time, RFS) ~risklevel,data = Risk)

pValue=1-pchisq(diff$chisq,df=1)

pValue=signif(pValue,4)

pValue=format(pValue, scientific = TRUE)

fit <- survfit(Surv(Time, RFS) ~ risklevel, data =Risk)

plot(fit, lwd=2,col=c("red","blue"),

xlab="Time (months)",

ylab="Recurrence-free survival",

mark.time=T)

text(30,0.1,paste("***P***value:", pValue,sep=""),col="black")

legend("topright",

c("High risk", "Low risk"),

lwd=2,

col=c("red","blue"))

**ROC曲线 Clinical**

**TCGA**

library(survivalROC)

setwd("D:\\科研论文\\科研学术\\生信研究\\LSCC\\IRLs\\数据分析\\ROC DCA")

dev<-read.csv("TCGA.csv")

cutoff<-60

Nom=survivalROC(Stime=dev$Time, status= dev$OS,

marker= dev$IRL.score, entry = NULL, predict.time=cutoff, cut.values =NULL, method = "KM", lambda = NULL, span = NULL, window ="symmetric")

plot(Nom$FP,Nom$TP,type="l",xlim=c(0,1),ylim=c(0,1),col="red",ylab="Sensitivity", xlab="1-Specificity",lwd=2)

abline(a=0,b=1,col="black",lwd=1.5)

Lnc=survivalROC(dev$Time, status= dev$OS,

marker= dev$Margin.status, entry = NULL, predict.time=cutoff, cut.values =NULL, method = "KM", lambda = NULL, span = NULL, window ="symmetric")

lines(Lnc$FP,Lnc$TP,type="l",xlim=c(0,1),ylim=c(0,1),col="blue",ylab="Sensitivity", xlab="1-Specificity",lwd=2)

Lc=survivalROC(dev$Time, status= dev$OS,

marker= dev$Lymphovascularinvasion, entry = NULL, predict.time=cutoff, cut.values =NULL, method = "KM", lambda = NULL, span = NULL, window ="symmetric")

lines(Lc$FP,Lc$TP,type="l",xlim=c(0,1),ylim=c(0,1),col="orange",ylab="Sensitivity", xlab="1-Specificity",lwd=2)

TNM=survivalROC(dev$Time, status= dev$OS,

marker= dev$Stage, entry = NULL, predict.time=cutoff, cut.values =NULL, method = "KM", lambda = NULL, span = NULL, window ="symmetric")

lines(TNM$FP,TNM$TP,type="l",xlim=c(0,1),ylim=c(0,1),col="#FFD700",ylab="Sensitivity", xlab="1-Specificity",lwd=2)

legend("bottomright",

c(paste("IRL score: ",round(Nom$AUC,3)), paste("Margin status: ",round(Lnc$AUC,3)), paste("Lymphovascular invasion: ",round(Lc$AUC,3)),

paste("TNM stage:",round(TNM$AUC,3))),col=c("red","blue","orange", "#FFD700"),lwd=2)

**GSE65858**

library(survivalROC)

setwd("D:\\科研论文\\科研学术\\生信研究\\LSCC\\IRLs\\数据分析\\ROC DCA")

dev<-read.csv("GSE65858.csv")

cutoff<-36

Nom=survivalROC(Stime=dev$Time, status= dev$OS,

marker= dev$IRL.score, entry = NULL, predict.time=cutoff, cut.values =NULL, method = "KM", lambda = NULL, span = NULL, window ="symmetric")

plot(Nom$FP,Nom$TP,type="l",xlim=c(0,1),ylim=c(0,1),col="red",ylab="Sensitivity", xlab="1-Specificity",lwd=2)

abline(a=0,b=1,col="black",lwd=1.5)

Lnc=survivalROC(dev$Time, status= dev$OS,

marker= dev$Margin.status, entry = NULL, predict.time=cutoff, cut.values =NULL, method = "KM", lambda = NULL, span = NULL, window ="symmetric")

lines(Lnc$FP,Lnc$TP,type="l",xlim=c(0,1),ylim=c(0,1),col="blue",ylab="Sensitivity", xlab="1-Specificity",lwd=2)

Lc=survivalROC(dev$Time, status= dev$OS,

marker= dev$Gender, entry = NULL, predict.time=cutoff, cut.values =NULL, method = "KM", lambda = NULL, span = NULL, window ="symmetric")

lines(Lc$FP,Lc$TP,type="l",xlim=c(0,1),ylim=c(0,1),col="orange",ylab="Sensitivity", xlab="1-Specificity",lwd=2)

TNM=survivalROC(dev$Time, status= dev$OS,

marker= dev$Stage, entry = NULL, predict.time=cutoff, cut.values =NULL, method = "KM", lambda = NULL, span = NULL, window ="symmetric")

lines(TNM$FP,TNM$TP,type="l",xlim=c(0,1),ylim=c(0,1),col="#FFD700",ylab="Sensitivity", xlab="1-Specificity",lwd=2)

legend("bottomright",

c(paste("IRL score: ",round(Nom$AUC,3)), paste("Age: ",round(Lnc$AUC,3)), paste("Sex: ",round(Lc$AUC,3)),

paste("TNM stage:",round(TNM$AUC,3))),col=c("red","blue","orangl", "#FFD700"),lwd=2)

**GSE65858**

library(survivalROC)

setwd("D:\\科研论文\\科研学术\\生信研究\\LSCC\\IRLs\\数据分析\\ROC DCA")

dev<-read.csv("GSE65858.csv")

cutoff<-36

Nom=survivalROC(Stime=dev$Time, status= dev$OS,

marker= dev$IRL.score, entry = NULL, predict.time=cutoff, cut.values =NULL, method = "KM", lambda = NULL, span = NULL, window ="symmetric")

plot(Nom$FP,Nom$TP,type="l",xlim=c(0,1),ylim=c(0,1),col="red",ylab="Sensitivity", xlab="1-Specificity",lwd=2)

abline(a=0,b=1,col="black",lwd=1.5)

Lnc=survivalROC(dev$Time, status= dev$OS,

marker= dev$Age, entry = NULL, predict.time=cutoff, cut.values =NULL, method = "KM", lambda = NULL, span = NULL, window ="symmetric")

lines(Lnc$FP,Lnc$TP,type="l",xlim=c(0,1),ylim=c(0,1),col="blue",ylab="Sensitivity", xlab="1-Specificity",lwd=2)

TNM=survivalROC(dev$Time, status= dev$OS,

marker= dev$Stage, entry = NULL, predict.time=cutoff, cut.values =NULL, method = "KM", lambda = NULL, span = NULL, window ="symmetric")

lines(TNM$FP,TNM$TP,type="l",xlim=c(0,1),ylim=c(0,1),col="#FFD700",ylab="Sensitivity", xlab="1-Specificity",lwd=2)

legend("bottomright",

c(paste("IRL score: ",round(Nom$AUC,3)), paste("Age: ",round(Lnc$AUC,3)), paste("TNM stage:",round(TNM$AUC,3))),col=c("red","blue", "#FFD700"),lwd=2)

**GSE27020**

library(survivalROC)

setwd("D:\\科研论文\\科研学术\\生信研究\\LSCC\\IRLs\\数据分析\\ROC DCA")

dev<-read.csv("GSE27020.csv")

cutoff<-60

Nom=survivalROC(Stime=dev$Time, status= dev$RFS,

marker= dev$IRL.score, entry = NULL, predict.time=cutoff, cut.values =NULL, method = "KM", lambda = NULL, span = NULL, window ="symmetric")

plot(Nom$FP,Nom$TP,type="l",xlim=c(0,1),ylim=c(0,1),col="red",ylab="Sensitivity", xlab="1-Specificity",lwd=2)

abline(a=0,b=1,col="black",lwd=1.5)

Lnc=survivalROC(dev$Time, status= dev$RFS,

marker= dev$Age, entry = NULL, predict.time=cutoff, cut.values =NULL, method = "KM", lambda = NULL, span = NULL, window ="symmetric")

lines(Lnc$FP,Lnc$TP,type="l",xlim=c(0,1),ylim=c(0,1),col="blue",ylab="Sensitivity", xlab="1-Specificity",lwd=2)

TNM=survivalROC(dev$Time, status= dev$RFS,

marker= dev$Stage, entry = NULL, predict.time=cutoff, cut.values =NULL, method = "KM", lambda = NULL, span = NULL, window ="symmetric")

lines(TNM$FP,TNM$TP,type="l",xlim=c(0,1),ylim=c(0,1),col="#FFD700",ylab="Sensitivity", xlab="1-Specificity",lwd=2)

legend("bottomright",

c(paste("IRL score: ",round(Nom$AUC,3)), paste("Age: ",round(Lnc$AUC,3)), paste("TNM stage:",round(TNM$AUC,3))),col=c("red","blue", "#FFD700"),lwd=2)

**DCA**

**TCGA**

source("stdca.R")

setwd("D:\\科研论文\\科研学术\\生信研究\\LSCC\\IRLs\\数据分析\\ROC DCA\\DCA")

dev<-read.csv("TCGA.csv")

head(dev)

library(survival)

#数据转成因子

Srv = Surv(dev$Time, dev$OS)

coxmod = coxph(Srv ~ BARX1_DT+KLHL7_DT+LINC02154, data=dev)

coxLnc = coxph(Srv ~ Margin.status,data=dev)

coxLc = coxph(Srv ~ Lymphovascularinvasion,data=dev)

coxTNM = coxph(Srv ~ Stage,data=dev)

#5年生存时间

dev$IRL.score = c(1-(summary(survfit(coxmod,newdata=dev), times=12)$surv))

dev$Margin.status =

c(1-(summary(survfit(coxLnc,newdata=dev),times=12)$surv))

dev$Lymphovascular.invasion =

c(1-(summary(survfit(coxLc,newdata=dev),times=12)$surv))

dev$TNM.stage = c(1-(summary(survfit(coxTNM,newdata=dev), times=12)$surv))

stdca(data=dev, outcome="OS", ttoutcome="Time", timepoint=12,

predictors=c("IRL.score","Margin.status","Lymphovascular.invasion", "TNM.stage"),xstop=1.0, smooth=TRUE)

setwd("D:\\科研论文\\科研学术\\生信研究\\LSCC\\IRLs\\数据分析\\ROC DCA\\DCA")

source("dca.R")

library(nricens)

library(rms)

library(foreign)

dev<-read.csv("TCGA.csv")

head(dev)

modelA <- glm(OS~BARX1_DT+KLHL7_DT+LINC02154, data = dev, family = binomial(link="logit"),x=TRUE)

summary(modelA)

dev$IRL_score<- predict(newdata=dev,modelA,"response")

modelB <- glm(OS ~Margin.status, data = dev, family = binomial(link="logit"),x=TRUE)

summary(modelB)

dev$Margin_status<- predict(newdata=dev,modelB,"response")

modelC <- glm(OS ~Lymphovascularinvasion, data = dev, family = binomial(link="logit"),x=TRUE)

summary(modelC)

dev$Lymphovascular_invasion<- predict(newdata=dev,modelC,"response")

modelD <- glm(OS ~Stage, data = dev, family = binomial(link="logit"),x=TRUE)

summary(modelD)

dev$TNM_stage<- predict(newdata=dev,modelD,"response")

#Decision Curve Analysis

dca(data=dev, outcome="OS", predictors=c("IRL_score", "Margin_status","Lymphovascular_invasion","TNM_stage"),smooth="TRUE", probability=c("TRUE", "TRUE","TRUE","TRUE"))

**GSE65858**

source("stdca.R")

setwd("D:\\科研论文\\科研学术\\生信研究\\LSCC\\IRLs\\数据分析\\ROC DCA\\DCA")

dev<-read.csv("GSE65858.csv")

head(dev)

library(survival)

#数据转成因子

Srv = Surv(dev$Time, dev$OS)

coxmod = coxph(Srv ~ BARX1_DT+KLHL7_DT+LINC02154, data=dev)

coxLc = coxph(Srv ~ Sex,data=dev)

coxLnc = coxph(Srv ~ Age,data=dev)

coxTNM = coxph(Srv ~ Stage,data=dev)

#5年生存时间

dev$IRL.score = c(1-(summary(survfit(coxmod,newdata=dev), times=18)$surv))

dev$Age =

c(1-(summary(survfit(coxLnc,newdata=dev),times=18)$surv))

dev$Sex =

c(1-(summary(survfit(coxLc,newdata=dev),times=18)$surv))

dev$TNM.stage = c(1-(summary(survfit(coxTNM,newdata=dev), times=18)$surv))

stdca(data=dev, outcome="OS", ttoutcome="Time", timepoint=18,

predictors=c("IRL.score","Age","Sex","TNM.stage"),xstop=1.0, smooth=TRUE)

**GSE27020**

source("stdca.R")

setwd("D:\\科研论文\\科研学术\\生信研究\\LSCC\\IRLs\\数据分析\\ROC DCA\\DCA")

dev<-read.csv("GSE27020.csv")

head(dev)

dev$Stage <-factor(dev$Stage,labels=c(' I', 'II',' III', 'IV'))

str(dev)

library(survival)

#数据转成因子

Srv = Surv(dev$Time, dev$RFS)

coxmod = coxph(Srv ~ BARX1_DT+KLHL7_DT+LINC02154, data=dev)

coxLnc = coxph(Srv ~ Age,data=dev)

coxTNM = coxph(Srv ~ Stage,data=dev)

#5年生存时间

dev$IRL.score = c(1-(summary(survfit(coxmod,newdata=dev), times=60)$surv))

dev$Age =

c(1-(summary(survfit(coxLnc,newdata=dev),times=60)$surv))

dev$TNM.stage = c(1-(summary(survfit(coxTNM,newdata=dev), times=60)$surv))

stdca(data=dev, outcome="OS", ttoutcome="Time", timepoint=60,

predictors=c("IRL.score","Age","TNM.stage"),xstop=1.0, smooth=TRUE)

setwd("D:\\科研论文\\科研学术\\生信研究\\LSCC\\IRLs\\数据分析\\ROC DCA\\DCA")

source("dca.R")

library(nricens)

library(rms)

library(foreign)

dev<-read.csv("GSE27020.csv")

head(dev)

modelA <- glm(RFS~BARX1_DT+KLHL7_DT+LINC02154, data = dev, family = binomial(link="logit"),x=TRUE)

summary(modelA)

dev$IRL_score<- predict(newdata=dev,modelA,"response")

modelB <- glm(OS ~Age, data = dev, family = binomial(link="logit"),x=TRUE)

summary(modelB)

dev$Age<- predict(newdata=dev,modelB,"response")

modelC <- glm(OS ~Stage, data = dev, family = binomial(link="logit"),x=TRUE)

summary(modelC)

dev$TNM_stage<- predict(newdata=dev,modelC,"response")

#Decision Curve Analysis

dca(data=dev, outcome="RFS", predictors=c("IRL_score", "Age","TNM_stage"),smooth="TRUE", probability=c("TRUE", "TRUE","TRUE"))

**ICI**

**CIBERSORT**

setwd("D:\\科研论文\\科研学术\\生信研究\\LSCC\\IRLs\\数据分析\\ICI\\TCGA\\CIBERSORT")

inputfile1="id.txt" #生存时间数据

inputfile2="CIBERSORT.txt" #差异基因表达数据

time_data<-read.table(inputfile1,header = T,sep = "\t",check.names = F)

geneEXP<-read.table(inputfile2,header = T,sep = "\t",check.names = F)

head(time_data)

head(geneEXP)

merger_data<-merge(time_data,geneEXP,by="id")

write.table(merger_data,"CIBERSORTx.txt",sep = "\t",row.names = F,quote = F)

setwd("D:\\科研论文\\科研学术\\生信研究\\LSCC\\IRLs\\数据分析\\ICI\\TCGA\\CIBERSORT")

library(vioplot) #引用包

library(limma)

rt=read.table("CIBERSORTx.txt",sep="\t",header=T,row.names=1,check.names=F) #读取输入文件

normal=48 #正常样品数目

tumor=46 #肿瘤样品数目

pdf("CIBERSORT.pdf",height=8,width=15) #保存图片的文件名称

par(las=1,mar=c(10,6,3,3))

x=c(1:ncol(rt))

y=c(1:ncol(rt))

plot(x,y,

xlim=c(0,63),ylim=c(min(rt),max(rt)+0.02),

main="",xlab="", ylab="Fraction",

pch=21,

col="white",

xaxt="n")

#对每个免疫细胞循环，绘制vioplot，正常用绿色表示，肿瘤用红色表示

for(i in 1:ncol(rt)){

normalData=rt[1:normal,i]

tumorData=rt[(normal+1):(normal+tumor),i]

vioplot(normalData,at=3*(i-1),lty=1,add = T,col = 'blue')

vioplot(tumorData,at=3*(i-1)+1,lty=1,add = T,col = 'red')

wilcoxTest=wilcox.test(normalData,tumorData)

p=round(wilcoxTest$p.value,3)

mx=max(c(normalData,tumorData))

lines(c(x=3*(i-1)+0.2,x=3*(i-1)+0.8),c(mx,mx))

text(x=3*(i-1)+0.5,y=mx+0.02,labels=ifelse(p<0.001,paste0("p<0.001"),paste0("p=",p)),cex = 0.8)

legend("topright",

c("IRL low-risk group", "IRL high-risk group "),

lwd=3,bty="n",cex=1,

col=c("blue","red"))

text(seq(1,64,3),-0.05,xpd = NA,labels=colnames(rt),cex = 1,srt = 45,pos=2)

}

dev.off()

**Immune Cell**

setwd("D:\\科研论文\\科研学术\\生信研究\\LSCC\\IRLs\\数据分析\\ICI\\TCGA\\ImmuneCell")

inputfile1="id.txt" #生存时间数据

inputfile2="ImmuneCell.txt" #差异基因表达数据

time_data<-read.table(inputfile1,header = T,sep = "\t",check.names = F)

geneEXP<-read.table(inputfile2,header = T,sep = "\t",check.names = F)

head(time_data)

head(geneEXP)

merger_data<-merge(time_data,geneEXP,by="id")

write.table(merger_data,"ImmuneCellx.txt",sep = "\t",row.names = F,quote = F)

library(vioplot) #引用包

setwd("D:\\科研论文\\科研学术\\生信研究\\LSCC\\IRLs\\数据分析\\ICI\\TCGA\\ImmuneCell")

#设置工作目录

normal=54 #正常样品数目

tumor=54 #肿瘤样品数目

rt=read.table("ImmuneCellx.txt",sep="\t",header=T,row.names=1,check.names=F) #读取输入文件

pdf("ImmuneCell.pdf",height=9,width=18) #保存图片的文件名称

par(las=1,mar=c(10,6,3,3))

x=c(1:ncol(rt))

y=c(1:ncol(rt))

plot(x,y,

xlim=c(0,82),ylim=c(min(rt),max(rt)+0.02),

main="",xlab="", ylab="Immune infiltration",

pch=21,

col="white",

xaxt="n")

#对每个免疫细胞循环，绘制vioplot，正常用蓝色表示，肿瘤用红色表示

for(i in 1:ncol(rt)){

normalData=rt[1:normal,i]

tumorData=rt[(normal+1):(normal+tumor),i]

vioplot(normalData,at=3*(i-1),lty=1,add = T,col = 'blue')

vioplot(tumorData,at=3*(i-1)+1,lty=1,add = T,col = 'red')

wilcoxTest=wilcox.test(normalData,tumorData)

p=round(wilcoxTest$p.value,3)

mx=max(c(normalData,tumorData))

lines(c(x=3*(i-1)+0.2,x=3*(i-1)+0.8),c(mx,mx))

text(x=3*(i-1)+0.5,y=mx+0.02,labels=ifelse(p<0.001,paste0("p<0.001"),paste0("p=",p)),cex = 0.8)

legend("topright",

c("IRL low-risk group", "IRL high-risk group "),

lwd=3,bty="n",cex=1,

col=c("blue","red"))

text(seq(1,82,3),-0.1,xpd = NA,labels=colnames(rt),cex = 1,srt = 45,pos=2)

}

dev.off()

**GEO**

setwd("D:\\科研论文\\科研学术\\生信研究\\LSCC\\IRLs\\数据分析\\ICI\\GEO")

inputfile1="id.txt" #生存时间数据

inputfile2="CIBERSORTx.csv" #差异基因表达数据

time_data<-read.table(inputfile1,header = T,sep = "\t",check.names = F)

geneEXP<-read.table(inputfile2,header = T,sep = ",",check.names = F)

head(time_data)

head(geneEXP)

merger_data<-merge(time_data,geneEXP,by="id")

write.table(merger_data,"CIBERSORT.txt",sep = "\t",row.names = F,quote = F)

library(vioplot) #引用包

setwd("D:\\科研论文\\科研学术\\生信研究\\LSCC\\IRLs\\数据分析\\ICI\\GEO")

#设置工作目录

normal=55 #正常样品数目

tumor=54 #肿瘤样品数目

rt=read.table("CIBERSORT.txt",sep="\t",header=T,row.names=1,check.names=F) #读取输入文件

pdf("CIBERSORT.pdf",height=9,width=18) #保存图片的文件名称

par(las=1,mar=c(10,6,3,3))

x=c(1:ncol(rt))

y=c(1:ncol(rt))

plot(x,y,

xlim=c(0,64),ylim=c(min(rt),max(rt)+0.02),

main="",xlab="", ylab="Fraction",

pch=21,

col="white",

xaxt="n")

#对每个免疫细胞循环，绘制vioplot，正常用蓝色表示，肿瘤用红色表示

for(i in 1:ncol(rt)){

normalData=rt[1:normal,i]

tumorData=rt[(normal+1):(normal+tumor),i]

vioplot(normalData,at=3*(i-1),lty=1,add = T,col = 'blue')

vioplot(tumorData,at=3*(i-1)+1,lty=1,add = T,col = 'red')

wilcoxTest=wilcox.test(normalData,tumorData)

p=round(wilcoxTest$p.value,3)

mx=max(c(normalData,tumorData))

lines(c(x=3*(i-1)+0.2,x=3*(i-1)+0.8),c(mx,mx))

text(x=3*(i-1)+0.5,y=mx+0.02,labels=ifelse(p<0.001,paste0("p<0.001"),paste0("p=",p)),cex = 0.8)

legend("topright",

c("IRL low-risk group", "IRL high-risk group "),

lwd=3,bty="n",cex=1,

col=c("blue","red"))

text(seq(1,64,3),-0.04,xpd = NA,labels=colnames(rt),cex = 1,srt = 45,pos=2)

}

dev.off()

library(ggpubr) #引用包

tciaFile="CD8.txt" #免疫治疗打分文件

scoreFile="Group.txt" #m6A打分分组文件

setwd("D:\\科研论文\\科研学术\\生信研究\\LSCC\\IRLs\\数据分析\\ICI\\GEO")

#读取免疫治疗打分文件

ips=read.table(tciaFile, header=T, sep="\t", check.names=F, row.names=1)

#读取m6A打分分组文件

score=read.table(scoreFile, header=T, sep="\t", check.names=F, row.names=1)

#合并数据

sameSample=intersect(row.names(ips), row.names(score))

ips=ips[sameSample, , drop=F]

score=score[sameSample, "group", drop=F]

data=cbind(ips, score)

#设置比较组

data$group=factor(data$group, levels=c("Low", "High"))

group=levels(factor(data$group))

comp=combn(group, 2)

my_comparisons=list()

for(i in 1:ncol(comp)){my_comparisons[[i]]<-comp[,i]}

#对免疫治疗打分进行循环,分别绘制小提琴图

for(i in colnames(data)[1:(ncol(data)-1)]){

rt=data[,c(i, "group")]

colnames(rt)=c("IPS", "group")

gg1=ggviolin(rt, x="group", y="IPS", fill = "group",

xlab="CD8 T cells", ylab="Fraction",

legend.title="CD8 T cells",

palette=c("#0066FF", "#FF0000"),

add = "boxplot", add.params = list(fill="white"))+

stat_compare_means(comparisons = my_comparisons,symnum.args=list(cutpoints = c(0, 0.001, 0.01, 0.05, 1), symbols = c("***", "**", "*", "ns")),label = "p.signif")

pdf(file=paste0(i, ".pdf"), width=7, height=7)

print(gg1)

dev.off()

}

**Cor**

library(reshape2)

library(ggpubr)

library(ggExtra)

library(pheatmap)

setwd("D:\\科研论文\\科研学术\\生信研究\\LSCC\\IRLs\\数据分析\\ICI\\GEO")

rt=read.table("Cor.txt", header=T, sep="\t", check.names=F, row.names=1)

#读取输入文件

#绘制基因与免疫细胞相关性的散点图

x=as.numeric(rt[,"IRL score"])

y=as.numeric(rt[,"T cells CD8"])

df1=as.data.frame(cbind(x,y))

p1=ggplot(df1, aes(x, y)) +

xlab("IRL score") +

ylab("CD8 T cells") +

geom_point() + geom_smooth(method="lm",formula = y ~ x) + theme_bw()+

stat_cor(method = 'spearman', aes(x =x, y =y))

p2=ggMarginal(p1, type="density", xparams=list(fill = "orange"), yparams=list(fill = "blue"))

p2

**TCGA Cor**

library(reshape2)

library(ggpubr)

library(ggExtra)

library(pheatmap)

setwd("D:\\科研论文\\科研学术\\生信研究\\LSCC\\IRLs\\数据分析\\ICI\\TCGA\\CIBERSORT")

rt=read.table("Cor.txt", header=T, sep="\t", check.names=F, row.names=1)

#读取输入文件

#绘制基因与免疫细胞相关性的散点图

x=as.numeric(rt[,"IRL score"])

y=as.numeric(rt[,"T cells CD8"])

df1=as.data.frame(cbind(x,y))

p1=ggplot(df1, aes(x, y)) +

xlab("IRL score") +

ylab("CD8 T cells") +

geom_point() + geom_smooth(method="lm",formula = y ~ x) + theme_bw()+

stat_cor(method = 'spearman', aes(x =x, y =y))

p2=ggMarginal(p1, type="density", xparams=list(fill = "orange"), yparams=list(fill = "blue"))

p2

library(reshape2)

library(ggpubr)

library(ggExtra)

library(pheatmap)

setwd("D:\\科研论文\\科研学术\\生信研究\\LSCC\\IRLs\\数据分析\\ICI\\TCGA\\ImmuneCell")

rt=read.table("Cor.txt", header=T, sep="\t", check.names=F, row.names=1)

#读取输入文件

#绘制基因与免疫细胞相关性的散点图

x=as.numeric(rt[,"IRL score"])

y=as.numeric(rt[,"Activated CD8 T cell"])

df1=as.data.frame(cbind(x,y))

p1=ggplot(df1, aes(x, y)) +

xlab("IRL score") +

ylab("Activated CD8 T cell") +

geom_point() + geom_smooth(method="lm",formula = y ~ x) + theme_bw()+

stat_cor(method = 'spearman', aes(x =x, y =y))

p2=ggMarginal(p1, type="density", xparams=list(fill = "orange"), yparams=list(fill = "blue"))

p2

**TCGA IG**

library(ggpubr) #引用包

tciaFile="Estimate.txt" #免疫治疗打分文件

scoreFile="id.txt" #m6A打分分组文件

setwd("D:\\科研论文\\科研学术\\生信研究\\LSCC\\IRLs\\数据分析\\IG\\TCGA")

#读取免疫治疗打分文件

ips=read.table(tciaFile, header=T, sep="\t", check.names=F, row.names=1)

#读取m6A打分分组文件

score=read.table(scoreFile, header=T, sep="\t", check.names=F, row.names=1)

#合并数据

sameSample=intersect(row.names(ips), row.names(score))

ips=ips[sameSample, , drop=F]

score=score[sameSample, "group", drop=F]

data=cbind(ips, score)

write.table(data,"IG.txt",sep = "\t",row.names = F,quote = F)

library(ggpubr) #引用包

tciaFile="IG.txt" #免疫治疗打分文件

setwd("D:\\科研论文\\科研学术\\生信研究\\LSCC\\IRLs\\数据分析\\IG\\TCGA")

#读取免疫治疗打分文件

data=read.table(tciaFile, header=T, sep="\t", check.names=T, row.names=1)

#设置比较组

data$group=factor(data$group, levels=c("Low", "High"))

group=levels(factor(data$group))

comp=combn(group, 2)

my_comparisons=list()

for(i in 1:ncol(comp)){my_comparisons[[i]]<-comp[,i]}

#对免疫治疗打分进行循环,分别绘制小提琴图

for(i in colnames(data)[1:(ncol(data)-1)]){

rt=data[,c(i, "group")]

colnames(rt)=c("IPS", "group")

gg1=ggviolin(rt, x="group", y="IPS", fill = "group",

xlab="IRL score", ylab=i,

legend.title="IRL score",

palette=c("#0066FF", "#FF0000"),

add = "boxplot", add.params = list(fill="white"))+

stat_compare_means(comparisons = my_comparisons,symnum.args=list(cutpoints = c(0, 0.001, 0.01, 0.05, 1), symbols = c("***", "**", "*", "ns")),label = "p.signif")

pdf(file=paste0(i, ".pdf"), width=7, height=7)

print(gg1)

dev.off()

}

**Cor**

setwd("D:\\科研论文\\科研学术\\生信研究\\LSCC\\IRLs\\数据分析\\IG\\TCGA")

inputfile1="id.txt" #生存时间数据

inputfile2="MSI.txt" #差异基因表达数据

time_data<-read.table(inputfile1,header = T,sep = "\t",check.names = F)

geneEXP<-read.table(inputfile2,header = T,sep = "\t",check.names = F)

head(time_data)

head(geneEXP)

merger_data<-merge(time_data,geneEXP,by="id")

write.table(merger_data,"MSIx.txt",sep = "\t",row.names = F,quote = F)

setwd("D:\\科研论文\\科研学术\\生信研究\\LSCC\\IRLs\\数据分析\\IG\\TCGA")

inputfile1="MSIx.txt" #生存时间数据

inputfile2="TMB.txt" #差异基因表达数据

time_data<-read.table(inputfile1,header = T,sep = "\t",check.names = F)

geneEXP<-read.table(inputfile2,header = T,sep = "\t",check.names = F)

head(time_data)

head(geneEXP)

merger_data<-merge(time_data,geneEXP,by="id")

write.table(merger_data,"MSI+TMB.txt",sep = "\t",row.names = F,quote = F)

library(reshape2)

library(ggpubr)

library(ggExtra)

library(pheatmap)

setwd("D:\\科研论文\\科研学术\\生信研究\\LSCC\\IRLs\\数据分析\\IG\\TCGA")

rt=read.table("MSI+TCGA.txt", header=T, sep="\t", check.names=F, row.names=1)

#读取输入文件

#绘制基因与免疫细胞相关性的散点图

x=as.numeric(rt[,"IRL score"])

y=as.numeric(rt[,"MSI"])

df1=as.data.frame(cbind(x,y))

p1=ggplot(df1, aes(x, y)) +

xlab("IRL score") +

ylab("MSI") +

geom_point() + geom_smooth(method="lm",formula = y ~ x) + theme_bw()+

stat_cor(method = 'spearman', aes(x =x, y =y))

p2=ggMarginal(p1, type="density", xparams=list(fill = "orange"), yparams=list(fill = "blue"))

p2

**GEO IG**

library(utils)

rforge <- "http://r-forge.r-project.org"

install.packages("estimate", repos=rforge, dependencies=TRUE)

library(limma)

library(estimate)

setwd("D:\\科研论文\\科研学术\\生信研究\\LSCC\\IRLs\\数据分析\\IG\\GEO") #设置工作目录

inputFile="geneMatrix.txt" #输入文件名字

#读取文件,并对输入文件整理

rt=read.table(inputFile,sep="\t",header=T,check.names=F)

rt=as.matrix(rt)

rownames(rt)=rt[,1]

exp=rt[,2:ncol(rt)]

dimnames=list(rownames(exp),colnames(exp))

data=matrix(as.numeric(as.matrix(exp)),nrow=nrow(exp),dimnames=dimnames)

data=avereps(data)

#运行Estimate包

filterCommonGenes(input.f="geneMatrix.txt",

output.f="CommonGenes.gct",

id="GeneSymbol")

estimateScore(input.ds = "CommonGenes.gct",

output.ds="EstimateScore.gct",

platform="illumina")

#输出每个样品的打分

scores=read.table("EstimateScore.gct",skip = 2,header = T)

rownames(scores)=scores[,1]

scores=t(scores[,3:ncol(scores)])

rownames(scores)=gsub("\\.","\\-",rownames(scores))

out=rbind(ID=colnames(scores),scores)

write.table(out,file="Estimate.txt",sep="\t",quote=F,col.names=F)

library(ggpubr) #引用包

tciaFile="Estimate.txt" #免疫治疗打分文件

scoreFile="id.txt" #m6A打分分组文件

setwd("D:\\科研论文\\科研学术\\生信研究\\LSCC\\IRLs\\数据分析\\IG\\GEO")

#读取免疫治疗打分文件

ips=read.table(tciaFile, header=T, sep="\t", check.names=F, row.names=1)

#读取m6A打分分组文件

score=read.table(scoreFile, header=T, sep="\t", check.names=F, row.names=1)

#合并数据

sameSample=intersect(row.names(ips), row.names(score))

ips=ips[sameSample, , drop=F]

score=score[sameSample, "group", drop=F]

data=cbind(ips, score)

write.table(data,"IG.txt",sep = "\t",row.names = T,quote = F)

library(ggpubr) #引用包

tciaFile="IG.txt" #免疫治疗打分文件

setwd("D:\\科研论文\\科研学术\\生信研究\\LSCC\\IRLs\\数据分析\\IG\\GEO")

#读取免疫治疗打分文件

data=read.table(tciaFile, header=T, sep="\t", check.names=F, row.names=1)

#设置比较组

data$group=factor(data$group, levels=c("Low", "High"))

group=levels(factor(data$group))

comp=combn(group, 2)

my_comparisons=list()

for(i in 1:ncol(comp)){my_comparisons[[i]]<-comp[,i]}

#对免疫治疗打分进行循环,分别绘制小提琴图

for(i in colnames(data)[1:(ncol(data)-1)]){

rt=data[,c(i, "group")]

colnames(rt)=c("IPS", "group")

gg1=ggviolin(rt, x="group", y="IPS", fill = "group",

xlab="IRL score", ylab=i,

legend.title="IRL score",

palette=c("#0066FF", "#FF0000"),

add = "boxplot", add.params = list(fill="white"))+

stat_compare_means(comparisons = my_comparisons,symnum.args=list(cutpoints = c(0, 0.001, 0.01, 0.05, 1), symbols = c("***", "**", "*", "ns")),label = "p.signif")

pdf(file=paste0(i, ".pdf"), width=7, height=7)

print(gg1)

dev.off()

}

**SP TCGA**

setwd("D:\\科研论文\\科研学术\\生信研究\\LSCC\\IRLs\\数据分析\\SP\\TCGA")

inputfile1="id.txt" #生存时间数据

inputfile2="ImmuneSP.txt" #差异基因表达数据

time_data<-read.table(inputfile1,header = T,sep = "\t",check.names = F)

geneEXP<-read.table(inputfile2,header = T,sep = "\t",check.names = F)

head(time_data)

head(geneEXP)

merger_data<-merge(time_data,geneEXP,by="id")

write.table(merger_data,"ImmuneSPx.txt",sep = "\t",row.names = F,quote = F)

setwd("D:\\科研论文\\科研学术\\生信研究\\LSCC\\IRLs\\数据分析\\SP\\TCGA")

library(vioplot) #引用包

library(limma)

rt=read.table("ImmuneSPx.txt",sep="\t",header=T,row.names=1,check.names=F) #读取输入文件

normal=55 #正常样品数目

tumor=54 #肿瘤样品数目

pdf("ImmuneSP.pdf",height=8,width=12) #保存图片的文件名称

par(las=1,mar=c(10,6,3,3))

x=c(1:ncol(rt))

y=c(1:ncol(rt))

plot(x,y,

xlim=c(0,50),ylim=c(min(rt),max(rt)+0.02),

main="",xlab="", ylab="Immune infiltration",

pch=21,

col="white",

xaxt="n")

#对每个免疫细胞循环，绘制vioplot，正常用绿色表示，肿瘤用红色表示

for(i in 1:ncol(rt)){

normalData=rt[1:normal,i]

tumorData=rt[(normal+1):(normal+tumor),i]

vioplot(normalData,at=3*(i-1),lty=1,add = T,col = 'green')

vioplot(tumorData,at=3*(i-1)+1,lty=1,add = T,col = 'red')

wilcoxTest=wilcox.test(normalData,tumorData)

p=round(wilcoxTest$p.value,3)

mx=max(c(normalData,tumorData))

lines(c(x=3*(i-1)+0.2,x=3*(i-1)+0.8),c(mx,mx))

text(x=3*(i-1)+0.5,y=mx+0.02,labels=ifelse(p<0.001,paste0("p<0.001"),paste0("p=",p)),cex = 0.8)

legend("topleft",

c("IRL low-risk group", "IRL high-risk group "),

lwd=3,bty="n",cex=1,

col=c("green","red"))

text(seq(1,50,3),-0.1,xpd = NA,labels=colnames(rt),cex = 1,srt = 45,pos=2)

}

dev.off()

**FMSB**

setwd("D:\\科研论文\\科研学术\\生信研究\\LSCC\\IRLs\\数据分析\\SP\\TCGA")

#设置工作目录

library(fmsb)

data=read.table("FmsbInput.txt",header=T,sep="\t",row.names=1,check.names=F) #读取输入文件

maxValue=ceiling(max(abs(data))*10)/10

data=rbind(rep(maxValue,ncol(data)),rep(-maxValue,ncol(data)),data)

#定义颜色

colors="#cc9999"

#定义显著性

corStat=read.table("CorStat.txt",header=T,sep="\t",row.names=1,check.names=F)

colnames(data)=paste0(colnames(data),corStat$sig)

#输出结果

pdf(file="FMSB.pdf",height=6,width=6)

radarchart( data, axistype=1 ,

pcol=colors, #设置颜色

plwd=2 , #线条粗线

plty=1, #虚线，实线

cglcol="grey", #背景线条颜色

cglty=1, #背景线条虚线，实线

caxislabels=seq(-maxValue,maxValue,maxValue/2), #坐标刻度

cglwd=1.2, #背景线条粗细

axislabcol="#cccc33", #刻度颜色

vlcex=0.8 #字体大小

)

dev.off()

**GEO ssGSEA**

library(GSVA)

library(limma)

library(GSEABase)

setwd("D:\\科研论文\\科研学术\\生信研究\\LSCC\\IRLs\\数据分析\\SP\\GEO")

#设置工作目录

inputFile="geneMatrix.txt" #输入文件

gmtFile="ImmuneSP.gmt" #GMT文件

#读取输入文件，并对输入文件处理

rt=read.table(inputFile,sep="\t",header=T,check.names=F)

rt=as.matrix(rt)

rownames(rt)=rt[,1]

exp=rt[,2:ncol(rt)]

dimnames=list(rownames(exp),colnames(exp))

mat=matrix(as.numeric(as.matrix(exp)),nrow=nrow(exp),dimnames=dimnames)

mat=avereps(mat)

mat=mat[rowMeans(mat)>0,]

geneSet=getGmt(gmtFile,

geneIdType=SymbolIdentifier())

#ssgsea分析

ssgseaScore=gsva(mat, geneSet, method='ssgsea', kcdf='Gaussian', abs.ranking=TRUE)

#定义ssGSEA score矫正函数

normalize=function(x){

return((x-min(x))/(max(x)-min(x)))}

#对ssGSEA score进行矫正

ssgseaOut=normalize(ssgseaScore)

ssgseaOut=rbind(id=colnames(ssgseaOut),ssgseaOut)

write.table(ssgseaOut,file="ssGSEA.txt",sep="\t",quote=F,col.names=F)

setwd("D:\\科研论文\\科研学术\\生信研究\\LSCC\\IRLs\\数据分析\\SP\\GEO")

inputfile1="id.txt" #生存时间数据

inputfile2="ssGSEA.txt" #差异基因表达数据

time_data<-read.table(inputfile1,header = T,sep = "\t",check.names = F)

geneEXP<-read.table(inputfile2,header = T,sep = "\t",check.names = F)

head(time_data)

head(geneEXP)

merger_data<-merge(time_data,geneEXP,by="id")

write.table(merger_data,"ssGSEAx.txt",sep = "\t",row.names = F,quote = F)

setwd("D:\\科研论文\\科研学术\\生信研究\\LSCC\\IRLs\\数据分析\\SP\\GEO")

library(vioplot) #引用包

library(limma)

rt=read.table("ssGSEAx.txt",sep="\t",header=T,row.names=1,check.names=F) #读取输入文件

normal=55 #正常样品数目

tumor=54 #肿瘤样品数目

pdf("ImmuneSP.pdf",height=8,width=12) #保存图片的文件名称

par(las=1,mar=c(10,6,3,3))

x=c(1:ncol(rt))

y=c(1:ncol(rt))

plot(x,y,

xlim=c(0,50),ylim=c(min(rt),max(rt)+0.02),

main="",xlab="", ylab="Immune infiltration",

pch=21,

col="white",

xaxt="n")

#对每个免疫细胞循环，绘制vioplot，正常用绿色表示，肿瘤用红色表示

for(i in 1:ncol(rt)){

normalData=rt[1:normal,i]

tumorData=rt[(normal+1):(normal+tumor),i]

vioplot(normalData,at=3*(i-1),lty=1,add = T,col = 'green')

vioplot(tumorData,at=3*(i-1)+1,lty=1,add = T,col = 'red')

wilcoxTest=wilcox.test(normalData,tumorData)

p=round(wilcoxTest$p.value,3)

mx=max(c(normalData,tumorData))

lines(c(x=3*(i-1)+0.2,x=3*(i-1)+0.8),c(mx,mx))

text(x=3*(i-1)+0.5,y=mx+0.02,labels=ifelse(p<0.001,paste0("p<0.001"),paste0("p=",p)),cex = 0.8)

legend("topright",

c("IRL low-risk group", "IRL high-risk group "),

lwd=3,bty="n",cex=1,

col=c("green","red"))

text(seq(1,50,3),-0.1,xpd = NA,labels=colnames(rt),cex = 1,srt = 45,pos=2)

}

dev.off()

**FMSB**

library(fmsb)

setwd("D:\\科研论文\\科研学术\\生信研究\\LSCC\\IRLs\\数据分析\\SP\\GEO")

#设置工作目录

data=read.table("FmsbInput.txt",header=T,sep="\t",row.names=1,check.names=F) #读取输入文件

maxValue=ceiling(max(abs(data))*10)/10

data=rbind(rep(maxValue,ncol(data)),rep(-maxValue,ncol(data)),data)

#定义颜色

colors="#00FFFF"

#定义显著性

corStat=read.table("CorStat.txt",header=T,sep="\t",row.names=1,check.names=F)

colnames(data)=paste0(colnames(data),corStat$sig)

#输出结果

pdf(file="FMSB.pdf",height=6,width=7)

radarchart( data, axistype=1 ,

pcol=colors, #设置颜色

plwd=2 , #线条粗线

plty=1, #虚线，实线

cglcol="grey", #背景线条颜色

cglty=1, #背景线条虚线，实线

caxislabels=seq(-maxValue,maxValue,maxValue/2), #坐标刻度

cglwd=1.2, #背景线条粗细

axislabcol="#CC33CC", #刻度颜色

vlcex=0.8 #字体大小

)

dev.off()

**TCGA Chemokines**

**Cor**

library(reshape2)

library(ggpubr)

library(ggExtra)

library(pheatmap)

setwd("D:\\科研论文\\科研学术\\生信研究\\LSCC\\IRLs\\数据分析\\Chemokines\\TCGA")

rt=read.table("Cor.txt", header=T, sep="\t", check.names=F, row.names=1)

#读取输入文件

#绘制基因与免疫细胞相关性的散点图

x=as.numeric(rt[,"IRL score"])

y=as.numeric(rt[,"CXCL10"])

df1=as.data.frame(cbind(x,y))

p1=ggplot(df1, aes(x, y)) +

xlab("IRL score") +

ylab("CXCL10") +

geom_point() + geom_smooth(method="lm",formula = y ~ x) + theme_bw()+

stat_cor(method = 'spearman', aes(x =x, y =y))

p2=ggMarginal(p1, type="density", xparams=list(fill = "orange"), yparams=list(fill = "blue"))

p2

**GEO Chemokines**

**Cor**

library(reshape2)

library(ggpubr)

library(ggExtra)

library(pheatmap)

setwd("D:\\科研论文\\科研学术\\生信研究\\LSCC\\IRLs\\数据分析\\Chemokines\\GEO")

rt=read.table("Cor.txt", header=T, sep="\t", check.names=F, row.names=1)

#读取输入文件

#绘制基因与免疫细胞相关性的散点图

x=as.numeric(rt[,"IRL score"])

y=as.numeric(rt[,"CXCL10"])

df1=as.data.frame(cbind(x,y))

p1=ggplot(df1, aes(x, y)) +

xlab("IRL score") +

ylab("CXCL10") +

geom_point() + geom_smooth(method="lm",formula = y ~ x) + theme_bw()+

stat_cor(method = 'spearman', aes(x =x, y =y))

p2=ggMarginal(p1, type="density", xparams=list(fill = "orange"), yparams=list(fill = "blue"))

p2

**TIDE**

library(plyr)

library(ggplot2)

library(ggpubr)

scoreFile="id.txt" #m6A打分文件

cliFile="TIDE.txt" #临床数据文件

trait="Responder" #临床性状

setwd("D:\\科研论文\\科研学术\\生信研究\\LSCC\\IRLs\\数据分析\\TIDE")

#读取输入文件

score=read.table(scoreFile, header=T, sep="\t", check.names=F, row.names=1)

cli=read.table(cliFile, header=T, sep="\t", check.names=F, row.names=1)

sameSample=intersect(row.names(score), row.names(cli))

rt=cbind(score[sameSample,,drop=F], cli[sameSample,,drop=F])

#定义临床性状的颜色

bioCol=c("#0066FF","#FF0000","#FF9900","#6E568C","#7CC767","#223D6C","#D20A13","#FFD121","#088247","#11AA4D")

bioCol=bioCol[1:length(unique(rt[,trait]))]

#统计高低评分组病人数目

rt1=rt[,c(trait, "group")]

colnames(rt1)=c("trait", "group")

df=as.data.frame(table(rt1))

#计算高低评分组的百分率

df=ddply(df, .(group), transform, percent = Freq/sum(Freq) * 100)

#百分比位置

df=ddply(df, .(group), transform, pos = (cumsum(Freq) - 0.5 * Freq))

df$label=paste0(sprintf("%.0f", df$percent), "%")

df$group=factor(df$group, levels=c("Low", "High"))

#绘制百分率图

p=ggplot(df, aes(x = factor(group), y = percent, fill = trait)) +

geom_bar(position = position_stack(), stat = "identity", width = .3) +

scale_fill_manual(values=bioCol)+

xlab("IRL score")+ ylab("Percent weight")+ guides(fill=guide_legend(title=trait))+

geom_text(aes(label = label), position = position_stack(vjust = 0.5), size = 3) +

#coord_flip()+

theme_bw()

pdf(file="TIDE.pdf", width=7, height=6)

print(p)

dev.off()

#设置比较组

rt2=rt[,c(trait, "IRL score")]

colnames(rt2)=c("trait", "IRL score")

type=levels(factor(rt2[,"trait"]))

comp=combn(type, 2)

my_comparisons=list()

for(i in 1:ncol(comp)){my_comparisons[[i]]<-comp[,i]}

#绘制箱线图

boxplot=ggboxplot(rt2, x="trait", y="IRL score", fill="trait",

xlab=trait,

ylab="IRL score",

legend.title=trait,width = .3,

palette=bioCol)+

stat_compare_means(comparisons=my_comparisons)

pdf(file="IRL score.pdf",width=7,height=6)

print(boxplot)

dev.off()

rt2=rt[,c(trait, "TMB")]

colnames(rt2)=c("trait", "TMB")

type=levels(factor(rt2[,"trait"]))

comp=combn(type, 2)

my_comparisons=list()

for(i in 1:ncol(comp)){my_comparisons[[i]]<-comp[,i]}

#绘制箱线图

boxplot=ggboxplot(rt2, x="trait", y="TMB", fill="trait",

xlab=trait,

ylab="TMB",

legend.title=trait,width = .3,

palette=bioCol)+

stat_compare_means(comparisons=my_comparisons)

pdf(file="TMB.pdf",width=7,height=6)

print(boxplot)

dev.off()

rt2=rt[,c(trait, "MSI")]

colnames(rt2)=c("trait", "MSI")

type=levels(factor(rt2[,"trait"]))

comp=combn(type, 2)

my_comparisons=list()

for(i in 1:ncol(comp)){my_comparisons[[i]]<-comp[,i]}

#绘制箱线图

boxplot=ggboxplot(rt2, x="trait", y="MSI", fill="trait",

xlab=trait,

ylab="MSI",

legend.title=trait,width = .3,

palette=bioCol)+

stat_compare_means(comparisons=my_comparisons)

pdf(file="MSI.pdf",width=7,height=6)

print(boxplot)

dev.off()

setwd("D:\\科研论文\\科研学术\\生信研究\\LSCC\\IRLs\\World Journal of Surgical Oncology")

library(survival)

rt=read.table("TCGA.txt",header=T,sep="\t",check.names=F)

outTab=data.frame()

for(gene in colnames(rt[,4:ncol(rt)])){

a=rt[,gene]<=median(rt[,gene])

diff=survdiff(Surv(Time, OS) ~a,data = rt)

pValue=1-pchisq(diff$chisq,df=1)

outTab=rbind(outTab,cbind(gene=gene,pvalue=pValue))

pValue=signif(pValue,4)

pValue=format(pValue, scientific = TRUE)

fit <- survfit(Surv(Time, OS) ~ a, data = rt)

summary(fit)

pdf(file=paste0(gene,".pdf"),width =7, height =7)

plot(fit,

lwd=2,

col=c("red","blue"),

xlab="Time (month)",

mark.time=T,

ylab="Overall survival",

main=paste("Survival curve (p=", pValue ,")",sep=""))

legend("topright",

c(paste(gene," High Expression",sep=""),

paste(gene," Low Expression",sep="") ),

lwd=2,

col=c("red","blue"))

dev.off()

}
